# Molecular Marker-Based Identification of Resistance to *Bipolaris sorokiniana* in Kazakh and Global Wheat Germplasm

**DOI:** 10.3390/biology15030244

**Published:** 2026-01-28

**Authors:** Ardak Bolatbekova, Alma Kokhmetova, Yerlan Dutbayev, Göksel Özer, Madina Kumarbayeva, Sholpan Bastaubayeva, Aidana Kharipzhanova, Makpal Nurzhuma, Zhenis Keishilov, Assiya Kokhmetova, Kanat Bakhytuly, Kanat Mukhametzhanov, Vladimir Tsygankov

**Affiliations:** 1 Department of Genetics and Breeding, Institute of Plant Biology and Biotechnology, Almaty 050040, Kazakhstan; yerlan.dutbayev@kaznaru.edu.kz (Y.D.); madina_kumar90@mail.ru (M.K.); aidankastar@gmail.com (A.K.); maki_87@mail.ru (M.N.); jeka-sayko@mail.ru (Z.K.); asia.k68@mail.ru (A.K.); kanat1499@gmail.com (K.B.); kanat.mukhametzhanov@bk.ru (K.M.); 2 Faculty of Agrobiology, Kazakh National Agrarian Research University, Almaty 050010, Kazakhstan; 3 Department of Plant Protection, Faculty of Agriculture, Bolu Abant Izzet Baysal University, Bolu 14030, Turkey; gokozer@gmail.com; 4 Kazakh Research Institute of Agriculture and Plant Growing, Almalybak 040909, Kazakhstan; sh.bastaubaeva@mail.ru; 5 Kazakh Research Institute of Horse Breeding and Forage Production, Aktobe 030014, Kazakhstan; zigan60@mail.ru

**Keywords:** wheat, *Bipolaris sorokiniana*, common root rot, disease resistance, leaf AUDPC (area under the disease progress curve), molecular markers, *Sb* genes

## Abstract

Diseases caused by *Bipolaris sorokiniana*, expressed as leaf spot blotch (SB) and common root rot (CRR), continue to limit spring wheat production, particularly in dry regions where yield losses may reach 35–40%. This study evaluated resistance to SB and CRR in fifty spring wheat genotypes at both seedling and adult plant stages and identified genetic sources of resistance using molecular markers linked to the *Sb1* and *Sb2* genes. Field trials were conducted in 2023 and 2024 in the Aktobe region under natural infection, artificial inoculation, and a fungicide-treated background. Based on leaf spot blotch severity quantified as the area under the disease progress curve (leaf AUDPC) under natural infection, nine genotypes displayed stable resistance across both years, while fungicide-treated plots revealed twenty-three resistant genotypes in 2023 and eighteen in 2024. Artificial inoculation identified five resistant lines in 2023 and one in 2024. Resistance to common root rot (CRR) was assessed independently based on subcrown internode (SCI) browning at the adult plant stage. Seedling assays confirmed consistent resistance in six genotypes, all of which carried *Sb1*, *Sb2*, or their combination. In total, *Sb* genes were detected in thirty-six of the fifty accessions, including genotypes from Kazakhstan, Russia, and several other countries. The presence of *Sb1* or *Sb2* was associated with reduced disease severity, particularly at the seedling stage. These findings identify valuable germplasm for breeding wheat with improved resistance to *B. sorokiniana* in Kazakhstan.

## Simple Summary

In arid and semi-arid regions, including Kazakhstan, wheat production is severely constrained by diseases caused by *Bipolaris sorokiniana*. This pathogen infects both root and leaf tissues and may reduce grain yield by more than 30%. The present study aimed to evaluate the resistance of fifty wheat breeding lines to *B. sorokiniana* under contrasting infection conditions. Field experiments were conducted over two growing seasons under natural infection, artificial inoculation, and fungicide-protected treatments. Disease severity was assessed to identify genotypes with stable resistance across years and environments. In parallel, seedling resistance was evaluated under controlled laboratory conditions to determine early-stage responses to the pathogen. Several wheat lines consistently exhibited low disease severity in the field and expressed resistance at the seedling stage. Genetic analysis revealed that many of these resistant lines carry heritable traits associated with enhanced tolerance to *B. sorokiniana*. The identified genotypes represent valuable genetic resources for wheat breeding programs aimed at improving disease resistance in dry agricultural regions.

## 1. Introduction

Wheat (*Triticum aestivum* L.) is one of the most important agricultural crops worldwide, ensuring global food security and economic stability. Wheat is one of the most important grain crops in Kazakhstan and plays a crucial role in ensuring national food security and economic stability. According to the Food and Agriculture Organization of the United Nations (FAO), global wheat production reached 781.38 million tons in 2023, highlighting its critical role in sustaining the world’s population [1,2]. Wheat is also the dominant cereal crop in Kazakhstan, where it plays a key role in national food security and agricultural production. Annually, approximately 12.8 million hectares are allocated to wheat cultivation, yielding 16–17 million tons of soft wheat (stat.gov.kz, accessed on 20 April 2023) [3]. However, this production level represents a 25% decrease compared with the previous year [1], largely due to the combined effects of abiotic stresses and plant diseases.

Among the most destructive wheat diseases is common root rot (CRR), caused primarily by *Bipolaris sorokiniana* and *Fusarium* spp. [4]. *B. sorokiniana* (teleomorph *Cochliobolus sativus*) is a highly versatile pathogen capable of infecting multiple plant organs, including seeds, roots, shoots, and leaves, and is responsible for diseases such as common root rot, seedling blight, head blight, black point, and leaf spot diseases of wheat and barley [5]. Under conditions favorable for disease development, yield losses associated with CRR may exceed 35–45% [6,7,8]. Al-Sadi [9] reported that *B. sorokiniana* is a major causal agent of black point, common root rot, and spot blotch diseases of wheat. In Kazakhstan, *B. sorokiniana* and *Fusarium* spp. are recognized as the principal causal agents of root rot, as confirmed by joint studies conducted in collaboration with CIMMYT experts [10,11]. The disease is particularly severe in regions with unstable climatic conditions and intensive agricultural practices, such as Kazakhstan, where it causes substantial economic losses [12,13,14].

In addition to root infections, *B. sorokiniana* is also the primary causal agent of spot blotch (SB), a foliar disease that develops on wheat leaves during later growth stages and negatively affects photosynthetic capacity, grain filling, and seed quality. Therefore, breeding spot-blotch-resistant wheat cultivars has been a priority in wheat-growing areas affected by this disease [8,15,16]. Another important CRR-associated pathogen, *Fusarium culmorum*, induces necrosis at the stem base, disrupts vascular tissue function, promotes plant lodging, and significantly reduces both yield and grain quality [17,18]. Comprehensive surveys have documented the global distribution and pathogenic diversity of *B. sorokiniana* and *Fusarium* spp. associated with CRR across major wheat-growing regions, including the USA, Canada, China, Turkey, and Azerbaijan [10,19,20,21].

Common root rot (CRR) and spot blotch (SB), although predominantly caused by the same pathogen (*B. sorokiniana*), represent two distinct disease manifestations that differ substantially in their infection processes, temporal dynamics, and phenotypic expression [5,22,23]. CRR primarily damages the subcrown internode and root tissues, impairing water and nutrient uptake, whereas SB develops on leaves, reducing photosynthetic efficiency during later growth stages [5,23]. Importantly, resistance to one disease phase does not necessarily confer resistance to the other [24,25].

Consequently, accurate assessment of wheat resistance to *B. sorokiniana* requires independent evaluation of foliar and root disease components. Foliar disease development can be reliably quantified using repeated assessments of leaf severity summarized as the area under the disease progress curve (leaf AUDPC), which reflects the temporal progression of spot blotch [26,27]. In contrast, CRR severity must be assessed destructively at a single time point based on visual evaluation of subcrown internode browning and root discoloration, as repeated measurements on the same plant are not feasible. Accordingly, in this study, resistance to *B. sorokiniana* was characterized by quantifying spot blotch resistance using leaf AUDPC and evaluating common root rot resistance using root severity measurements, ensuring a biologically consistent and methodologically sound differentiation between the two disease phenotypes.

The increasing prevalence and severity of wheat diseases are further exacerbated by climate change and intensive agricultural practices, which promote the emergence of new pathogen races and elevate disease pressure. Diseases such as rust, fusarium head blight, tan spot, septoria, and powdery mildew collectively pose a serious threat to wheat productivity and grain quality worldwide [28,29,30,31,32]. Therefore, timely disease detection and accurate phenotyping are essential for implementing effective management strategies and minimizing yield losses [33].

In addition to studying the pathogens of root rot, our previous research in Kazakhstan has focused on the genetic resistance of wheat to other important diseases, such as stem and leaf rust [34,35,36,37]. To broaden the understanding of biotic stresses in wheat, previous studies have addressed resistance to leaf spot diseases using field phenotyping and molecular approaches [38,39], including investigations of toxin-related resistance mechanisms in necrotrophic pathosystems involving *Pyrenophora tritici-repentis* [8,40,41].

The current study on CRR complements and extends this body of work by addressing another major biotic stress affecting wheat in Kazakhstan. By integrating pathogen diversity assessment, resistance genotyping, and field evaluation, this research aims to fill existing gaps and contribute to the development of wheat varieties with broad-spectrum and durable resistance.

The deployment of disease-resistant cultivars remains one of the most effective and environmentally sustainable strategies for protecting wheat from pathogenic threats. The use of molecular genetic markers enables rapid and accurate identification of resistance genes in breeding materials, thereby accelerating breeding programs through marker-assisted selection (MAS). A key advantage of MAS is its ability to detect resistance alleles derived from donor sources within elite germplasm, facilitating the efficient development of improved cultivars [42].

Early efforts to breed wheat resistant to root rot were constrained by the limited genetic variability for resistance within cultivated wheat germplasm. As noted previously [8], this limitation prompted the exploration of wild relatives, such as *Thinopyrum ponticum*, as alternative sources of resistance genes. Subsequent introgression of resistance loci from wild species into wheat has significantly advanced the genetic improvement of CRR resistance [43].

But the breeding process was complicated by the polygenic inheritance of resistance, its quantitative nature, and its strong correlation with other agronomically significant traits, such as yield and drought tolerance. For example, GWAS studies revealed that loci associated with resistance to *B. sorokiniana* are often linked to genes influencing root system morphology and maturation timing [44]. This genetic complexity necessitated the development of advanced marker-assisted selection techniques and precise phenotyping methods to minimize adverse effects on economically important traits. The genetic control of resistance to CRR is polygenic, requiring the analysis of complex quantitative trait loci (QTL) [45]. Unlike resistance to some other diseases, where single genes with major effects can often be identified, resistance to CRR involves multiple loci with smaller individual effects. This complexity poses challenges for breeding but also offers the advantage of potentially more durable and stable resistance against the pathogen over time.

Wheat resistance to root rot caused by *B. sorokiniana* presents a complex genetic challenge. Research has identified several genetic loci, such as the *Sb* genes, which play a crucial role in providing resistance to root rot. These genes have been localized on various chromosomes and are associated with resistance to other diseases, highlighting their potential for improving wheat breeding.

The genetic basis of resistance to this disease involves multiple quantitative trait loci (QTLs), which have been identified through bi-parental mapping populations. Four major resistance genes, designated as *Sb1*, *Sb2*, *Sb3*, and *Sb4*, have been mapped to specific chromosomes in wheat. These genes not only contribute to resistance against *B. sorokiniana* but also interact with other disease resistance mechanisms, such as those against leaf rust and stripe rust [46,47].

The *Sb1* gene, located on chromosome 7DS, is associated with the *Lr34* gene, which encodes an ATP-binding cassette (ABC) transporter providing broad-spectrum resistance to several foliar fungal diseases [46,48,49]. Although *Sb1* is co-located with the adult plant resistance gene Lr34, the contribution of this chromosomal region to disease responses at different growth stages remains a subject of discussion and is addressed in detail below. Another important gene is *Lr46*, linked to *Sb1* on chromosome 1BL, which confers resistance to leaf rust in adult plants and is also associated with stripe rust resistance [50]. The *Sb2* resistance locus has been mapped to chromosome 5BL, a genomic region that also harbors the *Tsn1* gene, which conditions sensitivity to the necrotrophic toxin ToxA produced by *Pyrenophora tritici-repentis* and *Parastagonospora nodorum*. This co-localization suggests potential genetic linkage rather than functional identity between resistance and toxin sensitivity loci [8,51]. *Sb3*, identified on chromosome 3BS, correlates with immune responses to *B. sorokiniana* [52], while *Sb4*, recently mapped to chromosome 4BL, offers comprehensive resistance by preventing infection on both leaves and sheaths [53].

Understanding the molecular mechanisms underlying these resistance genes is crucial for developing effective breeding strategies to enhance wheat resistance against *B. sorokiniana*. This knowledge will facilitate the creation of more resilient wheat varieties, ultimately mitigating the economic impacts of spot blotch disease on global wheat production.

The main objectives of this research included the following: (1) assessing the level of resistance to CRR in a diverse set of wheat accessions at both adult plant and seedling growth stages; (2) identifying genetic sources of resistance to *B. sorokiniana* by employing molecular markers linked to *Sb* genes; and (3) identifying wheat germplasm that demonstrate enhanced resistance in the conditions of the Aktobe region of Kazakhstan.

## 2. Materials and Methods

### 2.1. Experimental Materials

The collection comprises 50 spring wheat genotypes, including 10 cultivars and breeding lines from Kazakhstan, 27 cultivars from Russia, and 13 cultivars from other countries (Supplementary Table S1). This panel consists of 28 common wheat (*Triticum aestivum*) and 22 durum wheat (*Triticum durum*) entries. Notably, the cultivars included in this collection are widely utilized in breeding programs across Kazakhstan and Central Asian countries. To validate the disease assessment, the susceptible check cultivar Glenlea, the resistant check cultivar Salamouni, and the moderately resistant cultivar Pavon 76 were included as standard controls.

### 2.2. Fungal Isolates

Based on cultural and morphological characteristics, monospore isolates of *B. sorokiniana* were selected [54,55]. For this research, four *B. sorokiniana* isolates identified by [14] Kharipzhanova et al. (2025) as exhibiting the highest levels of aggressiveness were chosen for detailed investigation. Four single-spore isolates were used in this study. Isolates Kz48, Kz52, and Kz56 were obtained from the roots of the spring wheat variety Kazakhstan-10, and isolate Kz8 was obtained from the winter wheat line 231 [14]. Isolates *B. sorokiniana* were collected in the 2023–2024 crop season, near V. Almalybak in the Almaty region in the south-east of Kazakhstan. The species affiliation of the isolates used was previously confirmed by us based on species-specific PCR analyses using *COSA_F/R* primers [56]. This mix of four isolates was used in greenhouse and field trials.

Isolates *B. sorokiniana* were grown on SNA medium containing the following (g per 1 L): 1 g KH_2_PO_4_, 1 g KNO_3_, 0.5 g MgSO_4_·7H_2_O, 0.5 g KCl, 0.2 g dextrose, 0.2 g sucrose, and 20 g agar, to further stimulate sporulation. Isolates were grown at 23 °C under UV-light (12 h/day) for 12 to 14 days. Morphological characterization of isolates was conducted based on established criteria [54,55]. Following sporulation, conidial suspensions were prepared in sterile distilled water and plated onto water agar (WA) to isolate single spores, as described by [57] Shikur Gebremariam et al. (2018). All isolates were kept at 4 °C on PDA slants during the studies and stored at −80 °C in vials containing a 15:85 (*v*/*v*) glycerol: water solution for long-term storage.

The inoculum for the field trial with four isolates were propagated in the laboratory and applied to blocks of very susceptible varieties in the field, which were sown in early at the mid of April. When necessary, infection was promoted at least twice a week; these blocks provided heavily infected plant material as inoculum for the subsequent field screening [58].

### 2.3. Evaluation of CRR at the Seedling Stage

Greenhouse trials with the mix of four isolates, Kz48, Kz52, Kz56, and Kz8, were conducted at the Kazakh National Agrarian Research University (KazNARU), Almaty, Kazakhstan, in 2024, and set up in three replications as complete randomized blocks. Accessions were grown in plastic pots (7 × 7 × 7 cm) with three seeds per accession at 16–18 °C with alternating light/darkness periods of 12 h (5000 lux). When the second leaf was fully expanded, the plants were spray-inoculated with approximately 1 mL spore suspension/pot and immediately covered with plastic foil for 48 h to ensure 100% humidity. Inoculated plants were grown at 22–24 °C and 70% humidity for another 7 to 10 days until symptoms were clearly developed.

The infection response type was assessed on the second leaf of each plant following the scale of [59] Kokko et al. (1995). Resistance to *B. sorokiniana* at the seedling stage was assessed using a 0–5 rating scale as described by [59] Kokko et al. (1995). According to the ratings, the accessions were classified into the following groups: highly resistant (HR, 0–0.9), resistant (R, 1.0–1.9), moderately resistant (MR, 2.0–2.5), moderately susceptible (MS, 2.6–3.9), and susceptible (S, 4.0–5.0).

### 2.4. Field Evaluation

The wheat collection was evaluated at the Agricultural Experimental Station (AES), (50.2833° N 57.1667° E) in Central Kazakhstan, Aktobe, during the 2023–2024 cropping season. The experimental material was given a fertilizer dose of 60 kg/ha N and 30 kg/ha P_2_O_5_, and standard crop management practices were followed [60]. The fluctuations annually include 366.8 mm (2022–2023) and 582.3 mm (2023–2024) of inflation, so no irrigation was carried out during the growing season. The growing seasons were favorable for pathogen infection and disease development. Mean daily temperature and relative humidity showed similar trends in both years, although average temperatures were lower in 2023 than in the 2024 growing season. The average maximum air temperature for mid-May in 2023 and 2024 reached 21.61 °C and 16.44 °C, respectively. For May to July 2023, mean daily temperatures were 17.08 °C, 21.76 °C, and 24.44 °C, respectively, and in 2024, they were 13.12 °C, 22.89 °C, and 22.95 °C [61] (www.pogodaiklimat.ru/monitor.php accessed on 15 May 2025). Foliar disease (spot blotch) and root disease (common root rot, CRR) were evaluated as two independent disease phenotypes caused by *B. sorokiniana*, using distinct assessment methods appropriate for each disease phase. Spot blotch severity was assessed on leaves and quantified using leaf AUDPC, whereas CRR severity was evaluated independently based on subcrown internode (SCI) browning at a single time point. Standard methods [58] were used to determine the distribution and intensity of disease development. Wheat plants exhibiting disease symptoms were sampled from different plots across 15–20 fields in the Aktobe region. Sampling was conducted at the plant maturity stage, collecting root systems at a height of 20 cm from the soil surface. For each cultivar and line, fifty plants were selected based on visible disease symptoms, including leaf yellowing, root collar rot accompanied by root browning, discoloration of the lower internodes of the rhizome, as well as background damage of natural, fungicidal, or infectious origin. Plants were excavated with as much of the root system intact as possible and carefully washed prior to evaluation.

Wheat germplasm was evaluated for resistance to root rot in field trials under three conditions: (1) natural infection, (2) fungicide-treated background, and (3) artificial inoculation. For natural infection, field phenotyping was performed using wheat straw naturally infested with root rot pathogens as the inoculum source. For fungicide protection, seeds were treated with a registered fungicide (Raxil Ultra) prior to sowing, following the manufacturer’s guidelines; untreated control plots were included. For artificial inoculation, a suspension of *B. sorokiniana* was uniformly applied to the soil at sowing at a concentration of 1 × 10^5^–1 × 10^6^ conidia/mL. Additionally, infected straw was incorporated at 1 kg/m^2^ to enhance infection pressure. Control plots without inoculation were maintained.

Planting material was sown from 23 to 27 May and harvested from 23 to 31 August throughout the two years of the trial period. Accessions were in a completely randomized design with three replications and a plot size of 1 m^2^. The susceptible cultivar Glenlea was sown around the trials as a border and after every 10th accession to support B. sorokiniana infection. To increase infection, all accessions were spray inoculated at the seedling stage with a mix of four B. sorokiniana spot blotch isolates (No Kz48, Kz52, Kz56, and Kz8) with a spore concentration of 20,000 conidia/mL. The percentage of leaf area infected by spot blotch was assessed at three time points during the growing period. Disease incidence and severity in the field were assessed using the Zadoks growth scale, with severity scored when all entries were at or near GS 20–29 (tillering), GS 71–79 (milk), and GS 80–89 (dough development) [62]. Glenlea (susceptible) and Salamouni (resistant) served as control cultivars.

For each cultivar and line, fifty plants were selected based on visible disease symptoms, including leaf yellowing, root collar rot accompanied by root browning, discoloration of the lower internodes of the rhizome, as well as background damage of natural, fungicidal, or infectious origin. Plants were excavated with as much of the root system intact as possible and carefully washed prior to evaluation. The severity of damage was assessed based on discoloration of the roots and root collar.

Disease severity was determined by measuring the percentage of stem surface darkening at the subcrown internode (SCI), indicative of CRR symptoms. Disease severity was scored according to a 0–4 scale, where 0 indicated healthy plants, 1 corresponded to 1–10%—highly resistant (HR), 2 to 11–25%—resistant (R), 3 to 26–50%—moderately susceptible (MS), and 4 to 51–100%—susceptible (S) [58]. Disease severity (DS) was calculated using the formula proposed by McKinney [63] and Al-Tovi [64] (Equation (1)).

(1) $$DS=\frac{\sum d}{d_{max}\times n}$$

where *DS* represents disease severity, *d* is the severity score assigned to each plant, *d*_max_ is the maximum possible disease score, and *n* is the total number of evaluated plants per replicate.

Visual scoring was performed for each plot, using the double-digit scale (0–9) modified from Saari and Prescott’s severity scale for assessing wheat foliar diseases [65]. This visual scoring and subsequent leaf AUDPC calculation were applied exclusively to foliar disease (spot blotch) assessments. The first digit (D1) indicates disease progress in canopy height from the ground level, and the second digit (D2) stands for the severity or proportion of infected leaf area. The SB evaluation was repeated three times at weekly intervals, with the first performed at Zadoks’ GS63. For each evaluation, percentage disease severity was calculated with the following formula: % severity = (D1/9) × (D2/9) × 100. The area under the disease progress curve (leaf AUDPC) was calculated based on the three disease evaluations, using the formula:

$$AUDPC=\sum_{i=1}^{n}[\{(Y_{i}+Y_{\left(i+1\right)})/2\}\times {(t}_{\left(i+1\right)}-t_{i})]$$

where *Y_i_* is SB severity at time *t_i_*, *t*_(*i*+1)_—*t_i_* is the time interval (days) between two disease scores, n is the number of times when SB was recorded. Mean leaf AUDPC values from two replications in each year, as well as averaged values based on 2 years’ evaluation.

To determine genotypic and year variances among genotypes for traits of *B. sorokiniana* resistance, an analysis of variance (ANOVA) was performed using the R-Studio, R 4.3.3 version software according to the nonparametric Wilcoxson and Kruskal–Wallis tests. The significance of the calculations was assessed using the *p*-value [66], and coefficients of Pearson correlation were calculated using the mean values of the characters assessed [67].

Principal component analysis was performed, and biplots were prepared using R-studio software in R version 3.5.3 [68]. The broad-sense heritability index, which measures the percentage of phenotypic variation attributable to genetic determinants, was derived using the ANOVA results: h^2^ = SSg/SSt, where SSg is the sum of squares for a genotype, and SSt is the total sum of squares.

### 2.5. DNA Extraction and Molecular Screening of Sb Resistance Genes

Each genotype’s genomic DNA was extracted using the CTAB method from fresh leaves of individual plants at the two-leaf seedling stage [69]. The concentration and purity of the resulting preparation were measured using a NanoDrop One spectrophotometer (Thermo Fisher Scientific, Waltham, MA, USA). The DNA concentration for PCR was adjusted to 20 ng/µL. The presence of the *Sb* gene-associated loci linked to resistance to common root rot (CRR) was determined in the wheat genotype collection using linked molecular markers. Primers, linked to *Sb* genes, were employed according to certain approved protocols. The polymerase chain reaction (PCR) was conducted using the primers and annealing temperature settings that were specified for each *Sb* gene in the references (Supplementary Table S2). A Bio-Rad T100TM Thermal Cycler (Bio-Rad, Hercules, CA, USA) was used to conduct the PCR experiments. The PCR mixture (25 µL) contained 2.5 µL of genomic DNA (20 ng), 1 µL of each primer (1 pM/µL) (Sigma-Aldrich, St. Louis, MI, USA), 2.5 µL of dNTP mixture (2.5 mM, dCTP, dGTP, dTTP and dATP aqueous solution) (ZAO Sileks, Moscow, Russia), 2.5 µL MgCl2 (25 mM), 0.2 µL Taq polymerase (5 units µL) (ZAO Sileks, Moscow, Russia), 2.5 µL 10X PCR buffer and 12.8 µL ddH20. TBE buffer (45 mM Tris-borate, 1 mM EDTA, pH 8) was used to separate the amplification products, and ethidium bromide was added [70]. A 1000bp DNA ladder (Fermentas, Vilnius, Lithuania) was employed to gauge the size of the amplification fragment. The Gel Documentation System (Gel Doc XR+, BIO-RAD, Hercules, CA, USA) was used to visualize the results. Each sample underwent three separate tests.

## 3. Results

### 3.1. Genetic Variation and Trait Associations for Resistance to Bipolaris Sorokiniana in Wheat

The variance analysis of the leaf AUDPC parameter measured under field conditions revealed that the genotype factor remained the dominant source of variation (SS = 1530.56; F = 18.42; and *p* < 0.001), accounting for 87.83% of the total sum of squares. The broad-sense heritability was high (h^2^ = 0.82), indicating that leaf AUDPC values were largely genetically controlled. Despite being statistically significant (SS = 206.68; F = 3.85; and *p* < 0.05), the year effect contributed only 11.86%, while the residual variation was minimal (0.30%) (Table 1). Thus, the data confirm that genetic differences among genotypes are the primary determinant of leaf AUDPC, with environmental variation between years playing a comparatively minor role.

In contrast, for root disease severity assessed in the field based on SCI browning, the year effect was the main contributor to variation (SS = 38,456.6; F = 19.91; *p* < 0.001), explaining 96.9% of the total variation. Although the genotype effect was statistically significant (SS = 78,452.3; F = 5.241; *p* < 0.05), it accounted for only 2.55% of the total SS, with a moderate heritability estimate (hb^2^ = 0.55). The residual variance (0.49%) was low in terms of %SS, suggesting that most of the explainable variation was captured by the year component. These results emphasize that environmental conditions had a dominant influence on field-level disease severity, overshadowing genetic effects.

For seedling resistance to *B. sorokiniana* under artificial inoculation, the genotype factor was the primary source of variation (SS = 71.416; F = 15.62; and *p* < 0.001), contributing 31.56% to the total sum of squares, with hb^2^ = 0.63. The effect of replication was not significant (SS = 26.763 and F = 1.23), accounting for only 11.83% of the variation. The residual variance was substantial (55.29%), indicating considerable unexplained variability at the seedling stage. Nonetheless, the significant genotype effect and moderate heritability confirm that genetically determined differences among genotypes are expressed reliably under controlled inoculation conditions (Table 1).

The evaluation of genetic parameters across 50 spring wheat genotypes showed substantial phenotypic variation for all resistance-related traits (Table 2). Leaf AUDPC values measured under field conditions demonstrated very wide ranges in both years, indicating pronounced differences in genotypic susceptibility to *B. sorokiniana*: in 2023, values varied from 10.5 to 693.0 (mean 218.17, CV = 82.01%), while in 2024, they ranged from 60.0 to 892.0 (mean 380.95, CV = 70.47%). Seedling resistance also exhibited moderate but clear variability (0.8–4.6, mean 2.20, CV = 54.52%), confirming differentiated responses already at the early developmental stage. Field disease severity assessments across tillering, milk, and dough development stages revealed extremely high variability (CV > 80%), with scores spanning from nearly 0 to very high values, reflecting the coexistence of highly resistant and highly susceptible genotypes under natural infection conditions. Overall, the broad ranges, high standard deviations, and elevated coefficients of variation across traits underscore pronounced phenotypic diversity within the evaluated wheat set, providing a strong basis for selecting promising genotypes for breeding programs aimed at improving resistance to *B. sorokiniana*.

The consistently high coefficients of variation observed for both foliar and root resistance traits indicate substantial phenotypic diversity within the evaluated germplasm, providing a strong basis for multi-trait selection in breeding programs.

The correlation analysis revealed several strong and meaningful relationships among field disease severity scores, leaf AUDPC values, and seedling resistance to *B. sorokiniana* (Table 3). Disease severity at the tillering stage showed high positive correlations with severity at the milk (r = 0.762) and dough development stages (r = 0.737), indicating consistency in disease expression across early and later growth stages. Tillering severity was also moderately correlated with leaf AUDPC values, particularly with leaf AUDPC 2023 (r = 0.634), suggesting that early-season infection contributes substantially to overall disease development. Severity ratings at the milk stage demonstrated strong associations with dough development severity (r = 0.840) and were moderately correlated with AUDPC for both years (r = 0.666 for 2023 and r = 0.560 for 2024), confirming that mid-season assessments effectively capture cumulative disease pressure. At the dough development stage, disease severity remained moderately associated with leaf AUDPC 2023 (r = 0.625) and leaf AUDPC 2024 (r = 0.561), reinforcing the close relationship between late-season symptoms and total disease progression. The two leaf AUDPC datasets were highly correlated with each other (r = 0.948), reflecting strong consistency in disease patterns across years. In contrast, seedling resistance showed weak to moderate negative correlations with all field traits (r ranging from −0.184 to −0.403), indicating that resistance expressed at the seedling stage does not reliably predict field performance under natural infection. Collectively, these results highlight the importance of multi-stage field assessments for accurately characterizing resistance to *B. sorokiniana.*

### 3.2. Field Assessment of Wheat Genotypes for Resistance to Common Root Rot

Root disease severity caused by *B. sorokiniana* was independently assessed at the adult plant stage based on subcrown internode (SCI) browning, revealing substantial genotypic variation in resistance to common root rot (CRR) among the evaluated wheat genotypes (Supplementary Table S1). Adult plant resistance was assessed as SCI browning (root severity), whereas seedling resistance was evaluated using a leaf infection score under controlled inoculation.

**Figure 1:**
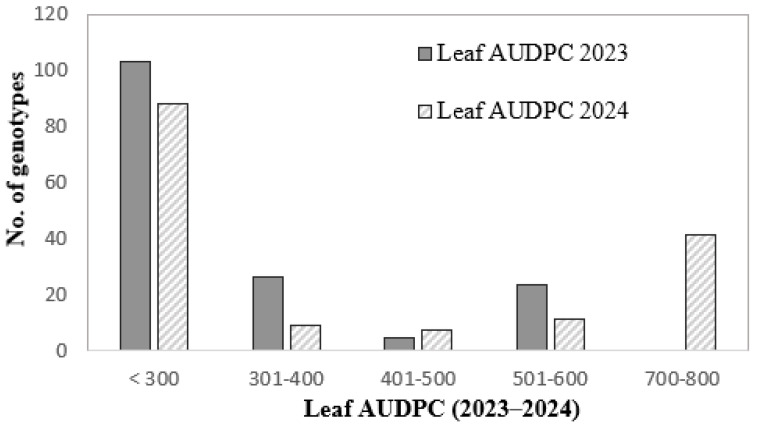
Distribution of wheat genotypes from Kazakhstan across leaf AUDPC classes for spot blotch resistance in the 2023 and 2024 field seasons.

The distribution of accessions concerning *B. sorokiniana* infection types in the adult plant stage (a) and seedling stage (b), with an indication of the standard error, is presented in Supplementary Table S1. CRR development varied greatly among the wheat entries. Most of the entries showed higher levels of resistance in the adult stage under field conditions than as seedlings (Supplementary Tables S1 and S3). The distribution of wheat genotypes across leaf AUDPC classes revealed pronounced differences between the 2023 and 2024 growing seasons (Figure 1). In 2023, most genotypes were classified within the low leaf AUDPC category (<300), indicating generally limited foliar disease development. Only a small proportion of genotypes fell into intermediate classes (301–500), and no entries exceeded leaf AUDPC values of 700. In contrast, the 2024 season showed a clear shift toward higher leaf AUDPC classes. While the <300 class remained represented, its relative contribution decreased, and a substantial number of genotypes shifted into higher disease categories (501–600 and 700–800). Notably, the 700–800 leaf AUDPC class, absent in 2023, became predominant in 2024, reflecting increased disease pressure under less favorable environmental conditions.

According to the field reactions under natural infection conditions in 2023, 18.0% of the evaluated wheat accessions exhibited high resistance to *B. sorokiniana*, 70.0% showed moderate resistance, and 12.0% were susceptible. Nine accessions (#363/k-43130; #377/k-44889-Yogui; #576/k-41350-Krasnokutka; #354/k-41295-Bankuti Garnet; #577/k-51744-Melanopus 2824; #445/Chelyaba 80; L-201m; #322/k-30949; and Kargala 9) demonstrated stable field resistance (AUDPC from 52 to 98). Resistance to *B. sorokiniana* isolates was associated with adult plant resistance to CRR in four genotypes (#363/k-43130; #577/k-51744-Melanopus 2824; #445/Chelyaba 80; and L-201m), which maintained high resistance under both greenhouse and field conditions. In the fungicide-treated trial in 2023, high resistance to CRR was recorded in 23 accessions (46.0%), moderate resistance in 24 accessions (48.0%), and susceptibility in 3 accessions (6.0%). Among the highly resistant group, seven genotypes (L-201m; #459/k-43285-Saratovskaya 35; #392/k-46619-Shenenskaya; #445/Chelyaba 80; #449/Orenburgskaya Yubileynaya, and #456/k-38531-Albidum 43) also exhibited resistance under greenhouse conditions (Supplementary Tables S1 and S4). Under artificial inoculation in 2023, high resistance was observed in five accessions (10.0%), moderate resistance in 23 accessions (46.0%), and susceptibility in 22 accessions (44.0%). The genotype L-201m showed consistent high resistance under both field and greenhouse conditions (Supplementary Tables S1 and S4).

Under natural infection conditions in 2024, 16.0% of the evaluated wheat accessions exhibited high resistance to *B. sorokiniana*, 70.0% showed moderate resistance, and 16.0% were susceptible. Eight accessions (#363/k-43130 (158); #377/k-44889-Yogui (178); #576/k-41350-Krasnokutka (180); #577/k-51744-Melanopus 2824 (186); #445/Chelyaba 80 (192); #449/Orenburgskaya Yubileynaya (196); #392/k-46619-Shenenskaya (198); and #459/k-43285-Saratovskaya 35 (198)) demonstrated consistently high field resistance. In six genotypes (#363/k-43130; #577/k-51744-Melanopus 2824; #445/Chelyaba 80; #449/Orenburgskaya Yubileynaya; #392/k-46619-Shenenskaya; and #459/k-43285-Saratovskaya 35), resistance to *B. sorokiniana* isolates was accompanied by high adult plant resistance to CRR, which was maintained under both greenhouse and field conditions (Supplementary Tables S1 and S4). Under a fungicide-treated background in 2024, 36.0% of the accessions (18 entries) were classified as highly resistant, 60.0% (30 entries) as moderately resistant, and 4.0% (2 entries) as susceptible. Six genotypes (L-201m; #392/k-46619-Shenenskaya; #445/Chelyaba 80; #449/Orenburgskaya Yubileynaya; #459/k-43285-Saratovskaya 35; #464/k-54045-Tselinnaya 21; #577/k-51744-Melanopus 2824; and #660/Line-2285-d.3) from the highly resistant category also displayed consistent resistance under greenhouse conditions (Supplementary Tables S1 and S2). Following artificial inoculation in 2024, high resistance was observed in only one accession (2.0%), moderate resistance in 23 accessions (46.0%), and susceptibility in 26 accessions (52.0%). Among these, the genotype #363/k-43130 maintained stable high resistance in both field and greenhouse experiments (Supplementary Tables S1 and S4).

The results of genotyping with markers linked to *Sb* resistance genes are presented in Supplementary Table S1. One or more *Sb* genes were detected in 36 of the 50 genotypes tested (72.0%). The molecular marker linked to *Sb1* (*csLV34*) amplified a 150 bp fragment in seven genotypes, representing 14% of all tested samples. The remaining 43 genotypes did not carry the *Sb1* gene. Among the *Sb1* carriers, seven genotypes (L-201m; #363/k-43130; #392/k-46619-Shenenskaya; #445/Chelyaba 80; #449/Orenburgskaya Yubileynaya; #456/k-38531-Albidum 43; and #459/k-43285-Saratovskaya 35) exhibited a highly resistant seedling response to a mixture of the most aggressive *B. sorokiniana* isolates under laboratory conditions (Supplementary Table S1).

The molecular marker linked to *Sb2* (*Xfcp623*) identified 15 genotypes (30%) carrying a null allele, which is indicative of the presence of the *Sb2* gene. The remaining 35 genotypes amplified a 380bp fragment, consistent with the absence of *Sb2*. Among *Sb2* carriers, six genotypes (L-201m, #392/k-46619-Shenenskaya, #445/Chelyaba 80, #449/Orenburgskaya Yubileynaya, #456/k-38531-Albidum 43, and #459/k-43285-Saratovskaya 35) also demonstrated a resistant seedling response to the isolate mixture (Supplementary Table S1).

Overall, six genotypes carrying both *Sb1* and *Sb2* genes displayed a highly resistant reaction to the mixture of the most aggressive *B. sorokiniana* isolates at the seedling stage.

**Figure 2:**
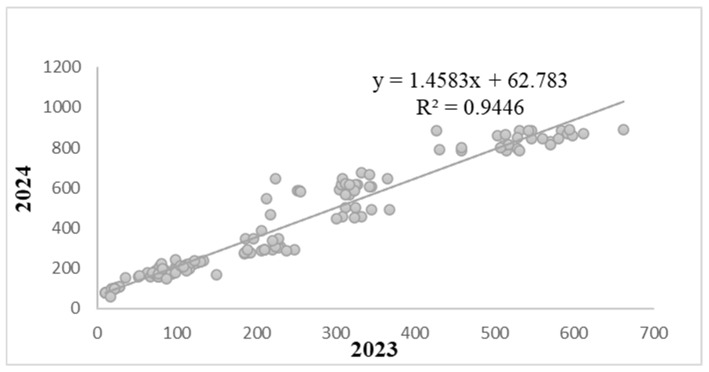
Resistance scores to *B. sorokiniana* at the adult plant stage of wheat genotypes with the corresponding leaf AUDPC values for the 2023 and 2024 field seasons.

Figure 2 displays the resistance scores together with the leaf AUDPC values for each genotype in 2023 and 2024, enabling an assessment of the consistency of field evaluations across years. A strong and statistically significant positive correlation was observed between the two seasons (r = 0.9446; *p* < 0.001). Linear regression analysis confirmed this high level of reproducibility, yielding a coefficient of determination of R^2^ = 0.9446, which indicates that nearly all variation in leaf AUDPC in 2024 was explained by the corresponding values in 2023. The regression equation (y = 1.4583x + 62.783) further shows that disease severity tended to be higher in 2024, as reflected by a more than proportional increase in leaf AUDPC relative to the previous year. The positive intercept suggests that even genotypes with low leaf AUDPC values in 2023 exhibited elevated baseline severity in 2024. Overall, these results demonstrate excellent year-to-year stability of leaf AUDPC and confirm the robustness of this parameter for quantifying resistance to *B. sorokiniana* under field conditions.

#### 3.2.1. Root Severity of Wheat Genotypes (SCI-Based Assessment)

Root disease severity caused by *B. sorokiniana* was independently evaluated based on subcrown internode (SCI) browning at the adult plant stage. Root severity varied widely among wheat genotypes, indicating substantial genotypic differentiation in resistance to common root rot (CRR) (Supplementary Table S1).

In common wheat genotypes, root disease severity ranged from 7.92% to 45.83%, representing responses from high resistance to pronounced susceptibility. Several genotypes exhibited low root severity (<10%), indicating effective restriction of pathogen development, whereas the majority showed moderate infection levels (20–30%), reflecting partial resistance. A subset of genotypes demonstrated high root severity (>40%), corresponding to strong susceptibility. Despite increasing disease pressure, genotype-dependent differences in root infection were consistently maintained, highlighting a strong genetic control of CRR response in common wheat.

Durum wheat genotypes also displayed substantial variation in root severity, with values ranging from 4.17% to 46.67%. Several entries, including control cultivars, exhibited low root infection levels (<10%), whereas most genotypes showed moderate severity (20–30%). Highly susceptible durum genotypes exceeded 40% root severity, indicating limited resistance to CRR. As observed in common wheat, genotype-specific differences were preserved across infection backgrounds.

Notably, discrepancies between foliar disease progression and root severity were observed for several genotypes, supporting the concept of partial independence between aboveground and belowground disease responses. These results emphasize the importance of separate assessment of root-related traits when evaluating resistance to common root rot.

#### 3.2.2. Correlation Between Root Severity and Leaf AUDPC

To evaluate the relationship between belowground and aboveground disease development caused by *B. sorokiniana*, a correlation analysis was performed between root disease severity assessed by subcrown internode (SCI) browning and foliar disease development quantified by leaf AUDPC values averaged across the 2023–2024 field seasons.

**Figure 3:**
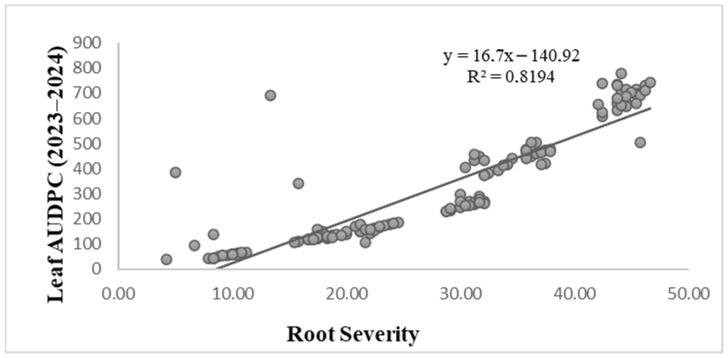
Correlation between root disease severity and foliar disease development (Leaf AUDPC) in wheat genotypes.

A statistically significant positive correlation was detected between root severity and leaf AUDPC (R^2^ = 0.8194; *p* < 0.001) (Figure 3). Linear regression analysis indicated that approximately 81.9% of the variation in foliar disease progression could be explained by differences in root infection severity among genotypes. As root severity increased, corresponding AUDPC values rose proportionally, demonstrating a strong overall association between the two disease components.

Despite this strong general trend, several genotypes deviated from the regression line, exhibiting either relatively low root severity combined with higher foliar AUDPC values or, conversely, increased root infection despite moderate foliar disease development. These deviations indicate that, although root- and leaf-disease responses are closely linked at the population level, genotype-specific differences in disease expression between belowground and aboveground tissues persist.

Overall, the results demonstrate a strong but not absolute correspondence between CRR severity and spot blotch development under field conditions, supporting the need for parallel evaluation of root and foliar traits when characterizing resistance to *B. sorokiniana*.

### 3.3. Seedling Resistance Assessment to B. sorokiniana

Resistance of 50 wheat accessions to *B. sorokiniana* at the seedling stage was evaluated under controlled conditions using a mixture of the most aggressive isolates (Kz48, Kz52, Kz56, and Kz8). Disease severity was assessed on seedling leaves using a 5-point scale according to Kokko et al. (1995) [59]. Based on these scores, the accessions were classified into five resistance categories: highly resistant (HR), resistant (R), moderately resistant (MR), moderately susceptible (MS), and susceptible (S).

The HR group comprised six accessions (12.0%), including L-201m, #392/k-46619-Shenenskaya, #445/Chelyaba 80, #449/Orenburgskaya Yubileynaya, #456/k-38531-Albidum 43, and #459/k-43285-Saratovskaya 35. The resistant category included 23 accessions (46.0%), while moderately resistant, moderately susceptible, and susceptible groups each contained 7 accessions (14.0%) (Supplementary Table S3 and Supplementary Figure S1). Overall, the majority of the evaluated germplasm exhibited resistance or moderate resistance at the seedling stage, indicating a substantial frequency of effective early-stage responses to *B. sorokiniana.*

**Figure 4:**
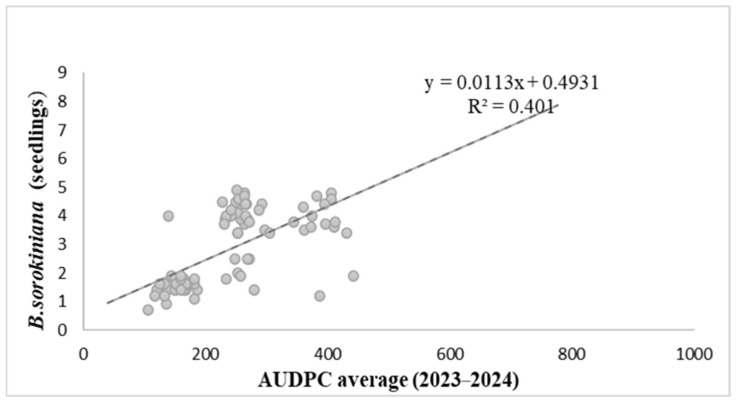
Relationship between resistance to *B. sorokiniana* at the seedling stage and adult plant resistance expressed as leaf AUDPC.

Figure 4 illustrates the relationship between seedling resistance scores and adult plant resistance expressed as mean leaf AUDPC values across the 2023–2024 field seasons. Linear regression analysis revealed a moderate positive association (R^2^ = 0.401; y = 0.0113x + 0.4931), indicating that approximately 40% of the variation in seedling infection severity was explained by cumulative foliar disease development under field conditions. Seedling infection scores varied widely among genotypes (approximately 1 to >7), and several accessions with comparable leaf AUDPC values exhibited contrasting responses at the seedling stage.

Despite the statistically significant relationship, a substantial scatter of data points around the regression line was observed (Figure 4), indicating pronounced genotypic variability. This wide dispersion suggests that seedling resistance explains only part of the variation in disease development under field conditions and does not represent a strong predictor of adult plant resistance.

These results demonstrate that resistance expressed at the seedling stage and adult plant resistance only partially overlap, supporting the conclusion that seedling assays capture only a limited fraction of the phenotypic variation observed under field conditions. This partial correspondence highlights the importance of combining seedling tests with multi-stage field evaluations for accurate characterization of resistance to *B. sorokiniana.*

### 3.4. Identification of Sb Resistance Genes Using Molecular Markers

The results of genotyping with markers linked to *Sb* resistance genes are given in Supplementary Table S1. *Sb1* was discovered in the common wheat line “Saar,” was mapped to chromosome 7DS, and is associated with the wheat leaf rust resistance gene *Lr34* [71]. It was shown that QTL *Sb1/Lr34* is associated with marker *csLV34* and other SSR markers near *Lr34* [48]. Another minor QTL linked to *Lr46* on chromosome 1BL was also identified from “Saar” [44].

Out of the 50 analyzed entries, the STS marker *csLV34* linked to *Sb1* amplified a 150 bp fragment in seven genotypes, including L-201m, #363/k-43130, #392/k-46619-Shenenskaya, #445/Chelyaba-80, #449/Orenburgskaya Yubileinaya, #456/k-38531-Albidum 43, and #459/k-43285-Saratovskaya 35, whereas the remaining 43 genotypes lacked the *Sb1*-associated allele (Supplementary Table S1).

**Figure 5:**
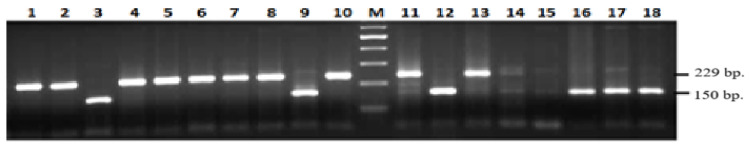
DNA amplification products for wheat cultivars and breeding lines using primers to the STS *csLV34* locus linked to the *Sb1* resistance gene. Lanes are as follows: 1—#347/k-38532-Albidum 24; 2—#352/k-40599-Saratovskaya 29; 3—L-201m; 4—#353/k-41218-Saratovskaya 28; 5—#354/k-41295-Bankuti Garnet; 6—#362/k-43109-Licofen; 7—#366/k-43878; 8—#377/k-44889-Yogui; 9—#363/k 43130; 10—#378/k-45151-Oktavia; M—molecular-weight marker (Gene-Ruler, 100bp DNA ladder); 11—#308/k-12927-delfi; 12—#392/k 46619-Shenenskaya; 13—#407/k-52321-WW16628; 14—#316/k-25761; 15—H_2_O (negative control); 16—#445/Chelyaba-80; 17—#449/Orenburgskaya Yubileinaya; and 18—Thatcher (*Lr34*) (positive control).

As an example, PCR amplification results for 16 representative genotypes are shown in Figure 5. Five genotypes (L-201m, #363/k-43130, #392/k-46619-Shenenskaya, #445/Chelyaba-80, and #449/Orenburgskaya Yubileinaya), together with the positive control Thatcher (*Lr34*), produced the expected 150 bp fragment, indicative of the *Sb1*-associated allele.

As an example, the PCR results for 16 genotypes are presented in Figure 5. Five genotypes (L-201m; #363/k 43130; #392/k 46619-Shenenskaya; #445/Chelyaba-80; and #449/Orenburgskaya Yubileinaya) and the positive control Thatcher (*Lr34*) had 150 bp fragments, indicative of the *Sb1* resistance gene.

The sizes of the bands for *Sb1* are 150 bp (lanes 3, 9, 12, 16, and 17—positive control). Fragments amplified by *csLV34* were separated in the 2% agarose gels. Lanes are as follows: 1—#347/k-38532-Albidum 24; 2—#352/k-40599-Saratovskaya 29; 3—L-201m; 4—#353/k-41218-Saratovskaya 28; 5—#354/k-41295-Bankuti Garnet; 6—#362/k-43109-Licofen; 7—#366/k-43878; 8—#377/k-44889-Yogui; 9—#363/k 43130; 10—#378/k-45151-Oktavia; M—molecular-weight marker (Gene-Ruler, 100bp DNA ladder); 11—#308/k-12927-delfi; 12—#392/k 46619-Shenenskaya; 13—#407/k-52321-WW16628; 14—#316/k-25761; 15—H_2_O (negative control); 16—#445/Chelyaba-80; 17—#449/Orenburgskaya Yubileinaya; and 18—Thatcher (*Lr34*) (positive control).

Eleven genotypes, including #347/k-38532-Albidum 24, #352/k-40599-Saratovskaya 29, #353/k-41218-Saratovskaya 28, #354/k-41295-Bankuti Garnet, #362/k-43109-Licofen, #366/k-43878, #377/k-44889-Yogui, #378/k-45151-Oktavia, #308/k-12927-delfi, #407/k-52321-WW16628, and #316/k-25761, showed no 150 bp amplification product, and were therefore classified as negative for the *Sb1*-associated allele (Figure 5).

Thus, verification using the STS marker *csLV34*, which is widely applied for genotyping the *Lr34/Sb1* region on chromosome 7DS, confirmed that none of the durum wheat accessions carried the *Sb1*-associated allele. Thus, all T. durum genotypes were unequivocally negative for *Sb1.*

The *Sb2* gene, conferring resistance to *B. sorokiniana*, is located on the long arm of chromosome 5BL. *Sb2* was identified in the wheat cultivar “Yangmai 6”. It was reported to be linked with the *Tsn1* gene, which confers host-selective sensitivity to the fungal toxin ToxA produced by *Pyrenophora tritici-repentis* [8,51]. QTL *Sb2/Tsn1* associated with marker *Xfcp623* linked with the *Tsn1* gene was used in our study.

PCR analysis using the *Xfcp623* marker identified 35 wheat genotypes carrying a null allele, indicative of the presence of the *Sb2* gene. These genotypes included L-201m; #303/k-12589; #316/k-25761; #317/k-28117-Blancar; #318/k-28130-Smena; #347/k-38532-Albidum 24; #352/k-40599-Saratovskaya 29; #353/k-41218-Saratovskaya 28; #362/k-43109-Licofen; #392/k-46619-Shenenskaya; #407/k-52321-WW16628; #445/Chelyaba 80; #449/Orenburgskaya Yubileynaya; #450/Silach; #456/k-38531-Albidum 43; #459/k-43285-Saratovskaya 35; #464/k-54045-Tselinnaya 21; L-248/258; #506/k-64718-Gord. 1739; #507/k-64721-Gord. 1732; #508/k-64723-Leucurum 1751; #517/Seymour 16; #523/Kostanayskaya 15; #524/Gordeiforme 1790; #527/Bezenchukskay 139; #531/Gordeiforme-910; #553/Gordeiforme-00-171-4; #567/Tselinogradskaya 75; #573/k-64967-Orenburgskaya 21; #577/k-51744-Melanopus 2824; #581/Prinadur; #643/Gordeiforme-2441; #644/Gordeiforme-2246; #655/G-13-62-2; and #660/Line-2285-d.3. The remaining 15 genotypes amplified a 380bp fragment, consistent with the absence of *Sb2.*

**Figure 6:**
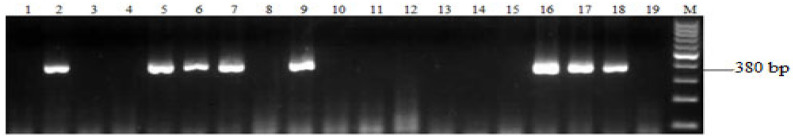
DNA amplification products for wheat cultivars and breeding lines obtained using primers for the SSR *Xfcp-623* locus linked to the *Sb2* resistance gene. The arrows indicate the 380bp band corresponding to *Sb2*-carrying germplasm (lanes 1, 3, 4, 8, 10, 11, 12, 13, 14, 15, and 19—positive control). PCR fragments amplified by *Xfcp-623* were separated on a 2% agarose gel. Lane 1—#392/k-46619-Shenenskaya; 2—#306/k-17172-Salamush; 3—L-201m; 4—#303/k-12589; 5—#308/k-12927-delfi; 6—#322/k-30949; 7—#324/k-31833; 8—#316/k-25761; 9—#354/k-41295-Bankuti Garnet; 10—#317/k-28117-Blancar; 11—#318/k-28130-Smena; 12—#347/k-38532-Albidum 24; 13—#352/k-40599-Saratovskaya 29; 14—#353/k-41218-Saratovskaya 28; 15—#362/k-43109-Licofen; 16—#363/k-43130; 17—#366/k-43878; 18—Glenlea (negative control); 19—Salamouni (positive control); and M—molecular-weight marker (GeneRuler, 100bp DNA ladder).

PCR results for 18 wheat genotypes, as an example, are presented in Figure 6. Among these, ten genotypes exhibited a null allele amplification, indicative of the presence of the *Sb2* gene: #392/k-46619-Shenenskaya; L-201m; #303/k-12589; #316/k-25761; #317/k-28117-Blancar; #318/k-28130-Smena; #347/k-38532-Albidum 24; #352/k-40599-Saratovskaya 29; #353/k-41218-Saratovskaya 28; and #362/k-43109-Licofen. The *Sb2* gene confers insensitivity to the root rot pathogen *B. sorokiniana*. The remaining genotypes amplified a 380bp fragment, consistent with the absence of *Sb2* and thus associated with susceptibility to CRR. Representative susceptible genotypes include #306/k-17172-Salamush; #308/k-12927-delfi; #322/k-30949; #324/k-31833; #354/k-41295-Bankuti Garnet; #363/k-43130; and #366/k-43878 (Figure 4).

So, one or two genes were detected in 36 of the 50 genotypes tested (72,0%) (Supplementary Table S1). In total, approximately 14.0% of all 50 entries assayed with STS markers in this study were predicted to possess *Sb1*. As for *Sb2*, approximately 70.0% of all 50 entries assayed with SSR markers in this study were predicted to possess *Sb2.* Six wheat genotypes simultaneously carried two *Sb* genes (L-201m, #392/k-46619-Shenenskaya, #445/Chelyaba 80, #449/Orenburgskaya Yubileynaya, #456/k-38531-Albidum 43, and #459/k-43285-Saratovskaya 35).

An analysis of the distribution of dependent variables among wheat samples differentiated by the presence or absence of the *B. sorokiniana* resistance genes *Sb1* and *Sb2* was performed (Supplementary Figure S2). To evaluate the impact of these genes on CRR severity, the resistance levels of carriers and non-carriers were compared using a non-parametric statistical approach. Specifically, the Kruskal–Wallis test was applied to examine differences in disease severity among groups defined by their *Sb* gene composition. The table summarizes the effect of the *Sb* resistance genes on seedling infection severity following inoculation with *B. sorokiniana*. Genotypes lacking both genes exhibited the highest infection level (3.92–3.95), confirming their pronounced susceptibility. The presence of either *Sb1* (≈1.40) or *Sb2* (≈1.60) markedly reduced the severity of infection, demonstrating the substantial contribution of these loci to early-stage resistance. The lowest infection score was observed in genotypes carrying both *Sb1* and *Sb2* (≈0.83–1.0), suggesting a potential additive or synergistic effect. The differences among all groups were statistically significant (*p* < 0.01), underscoring the importance of *Sb* genes in mediating seedling resistance to *B. sorokiniana*. However, despite the lower mean severity observed in *Sb1* + *Sb2* carriers, the combined presence of both genes did not lead to a statistically significant additional reduction compared to genotypes harboring *Sb1* or *Sb2* individually, indicating that each gene independently confers a comparable level of protection (Supplementary Figure S2).

Descriptive statistical parameters of seedling infection caused by *B. sorokiniana* revealed a broad phenotypic range, with values varying from 0.00 to 38.00, indicating substantial heterogeneity among the evaluated genotypes. The mean infection level (11.79) was comparable to the median (11.00), suggesting a moderately symmetrical distribution of the data, although the presence of high maximum values indicates a slight right-skewed tendency. The interquartile range was wide (Q1 = 3.00; Q3 = 17.25), demonstrating considerable variability within the central portion of the dataset: while 25% of seedlings exhibited minimal infection, the upper quartile represented genotypes with markedly elevated susceptibility. These results highlight pronounced phenotypic differentiation in pathogen response and underscore the relevance of further genetic and physiological investigations aimed at identifying wheat genotypes with enhanced resistance to *B. sorokiniana* (Supplementary Figure S3).

Generally, the presence of *Sb* genes was associated with increased resistance of wheat genotypes at the seedling stage. The presence of the *Sb1* gene in seven wheat genotypes, including L-201m, #363/k-43130, #392/k-46619-Shenenskaya, #445/Chelyaba 80, #449/Orenburgskaya Yubileynaya, #456/k-38531-Albidum 43, and #459/k-43285-Saratovskaya 35, was correlated with high resistance to CRR at the seedling stage. Likewise, six genotypes carrying the *Sb2* gene, including L-201m, #392/k-46619-Shenenskaya, #445/Chelyaba 80, #449/Orenburgskaya Yubileynaya, #456/k-38531-Albidum 43, and #459/k-43285-Saratovskaya 35, showed increased CRR resistance during the seedling stage. In field conditions, the impact of *Sb* genes on enhanced adult plant resistance was less pronounced.

The general variable showed that the population distribution between the groups falls within the following range: Min.—0.00; first Qu—3.00; Median—11.00; Mean—11.79; third Qu.—17.25; and Max.—38.00 (Supplementary Figure S3).

Thus, *Sb* genes have been identified in 36 wheat genotypes. Our analyses suggest that the correlation between the presence of *Sb* genes in these 36 entries and resistance is supported by the provided seedling resistance data, but not supported by data on field resistance.

## 4. Discussion

Resistance to common root rot (CRR), caused by *B. sorokiniana*, remains a major challenge for wheat production due to the complex epidemiology of the disease and the strong influence of environmental factors on its development [8,9,72,73]. In this study, we assessed the genetic variation in resistance to *Bipolaris sorokiniana* with a particular focus on the contribution of the *Sb1* and *Sb2* resistance genes to seedling and adult plant resistance, against the backdrop of the well-documented global and regional importance of this pathogen as a major production constraint [44,74,75].

Common root rot is especially damaging in arid wheat-growing areas, including the Aktobe Region of Kazakhstan, where the field trials were conducted. The pathogen thrives under high temperatures and moisture deficit typical in these environments [13,44,76]. In southern and western parts of the country, infection levels commonly reach 60–80%, and yield losses may approach 40% [14]. Because *B. sorokiniana* can persist in seeds and crop residues, it forms a long-term inoculum reservoir in the soil [9]. These conditions demonstrate the need to identify reliable sources of CRR resistance within Kazakh wheat germplasm.

Analysis of variance indicated that genotype was the dominant factor influencing leaf AUDPC, explaining nearly 88% of the total variation. The high broad-sense heritability (hb^2^ = 0.82) aligns with earlier findings that cumulative disease progress is primarily under genetic control and that leaf AUDPC is a stable indicator of quantitative resistance across environments [44,46]. In contrast, single-date severity scores were strongly affected by the year (96.9%), which underscores the sensitivity of adult plant responses to fluctuations in rainfall, temperature, and soil moisture [8,9]. Similar patterns, in which leaf AUDPC remains more stable than discrete severity ratings, have been documented in previous multi-environment studies on CRR [75]. The strong correlation of leaf AUDPC across years (r = 0.948) further supports the stability of genotype rankings despite pronounced interannual environmental variation.

Field surveys in Kazakhstan have shown a consistently high prevalence of CRR, with up to 80% of inspected fields affected, and demonstrated that disease severity is strongly associated with arid climatic conditions. Earlier studies clarified the relationships between disease intensity and agroecological factors, which enabled phytosanitary zoning of major wheat-growing regions [76,77,78,79].

The wide phenotypic variation observed for seedling resistance, field severity, and leaf AUDPC indicates substantial diversity within the tested germplasm, which agrees with findings from previous investigations of international wheat panels [8,72,75]. High coefficients of variation, often exceeding 80%, and broad ranges of disease reactions suggest the presence of clearly contrasting genotypes. Such diversity is essential for identifying donors of effective resistance and for uncovering additional loci contributing to CRR resistance. Strong positive correlations among severity scores at the tillering, milk, and dough stages (r = 0.737–0.840) indicate that susceptibility and resistance tendencies are relatively stable throughout development. The moderate to strong associations of these stage-specific scores with leaf AUDPC, particularly at the milk stage, confirm that mid-season assessments capture key components of cumulative disease progress, which is consistent with previous reports [72,75].

In contrast, the relationship between seedling resistance and field performance was notably weak. Seedling resistance showed only negative, weak-to-moderate correlations with adult plant traits (−0.184 to −0.403), and regression analysis (R^2^ = 0.408) confirmed its limited predictive value. These observations are consistent with earlier findings that seedling-stage resistance and adult plant resistance often only partially overlap due to differences in physiology, soil–root interactions, and variability in pathogen pressure under field conditions [9,73,80]. Therefore, relying solely on seedling assays may result in the exclusion of genotypes that express strong resistance at maturity. This discrepancy reflects biological differences between controlled seedling assays and complex field environments, where root–soil–climate interactions and cumulative infection pressure play a critical role. Our findings highlight the need for integrated phenotyping that combines seedling tests with multi-stage and multi-year field evaluations to accurately identify germplasm with robust CRR resistance.

Although a statistically significant association was detected between seedling resistance and adult plant resistance, the coefficient of determination and the wide dispersion of data points indicate that seedling resistance is not a strong predictor of field performance. This variability likely reflects the complex and quantitative nature of resistance to common root rot, which is influenced by developmental stage, environmental conditions, and multiple genetic factors that are predominantly expressed at later growth stages. Consequently, while seedling assays provide useful preliminary information, reliable identification of resistant genotypes requires multi-year field evaluations, underscoring the importance of adult plant assessments in resistance screening programs.

Comparative analysis of common (*Triticum aestivum* L.) and durum wheat (*Triticum durum* Desf.) revealed broadly similar patterns of root response to disease, characterized by substantial genotypic variability in both species. Root disease severity ranged from 7.92 to 45.83% in common wheat and from 4.17 to 46.67% in durum wheat, indicating that neither species exhibited consistently superior resistance at the root level. In both wheat types, the majority of genotypes displayed moderate root infection levels (20–30%), whereas a subset exhibited high susceptibility (>40%) under increased disease pressure.

Notably, the durum wheat collection included several genotypes with very low root disease severity (<10%), suggesting the presence of effective root resistance in specific genetic backgrounds. However, highly susceptible reactions were also observed in durum wheat, comparable to those detected in common wheat. Discrepancies between foliar disease development and root disease severity were evident in several genotypes, supporting the concept of partial independence between aboveground and belowground disease responses.

These findings emphasize that resistance to common root rot cannot be reliably inferred from foliar symptom expression alone and highlight the importance of independent assessment of root-related traits in breeding programs aimed at improving resistance in both common and durum wheat.

Earlier research in Kazakhstan has focused on elucidating the genetic basis of wheat resistance to root and foliar diseases and on developing molecular tools for diagnosis and marker-assisted selection [13,76]. Marker-assisted approaches previously applied to wheat rust resistance demonstrated the effectiveness of molecular screening for complex disease traits and provided a methodological framework [81] applicable to studies of resistance to leaf spots and other pathogens, including *B. sorokiniana* [14,82]. Consistent with these efforts, our molecular screening showed a wide distribution of *Sb* genes across the studied genotypes: 72% carried *Sb1*, *Sb2*, or both. *Sb1* occurred at a relatively low frequency (14%), whereas *Sb2* was present in 70% of the entries, a pattern similar to earlier reports describing its prevalence in South Asian and Eurasian wheat germplasm [44,48,72]. Seedling infection data confirmed that the presence of *Sb1* or *Sb2* reduced disease severity (≈1.4–1.6), while genotypes lacking both genes were highly susceptible (≈3.9–4.0). These trends agree with previous findings that mapped *Sb1* on chromosome 7DS near *Lr34* [48] and *Sb2* on chromosome 5BL associated with *Tsn1* [8,51], both of which contribute to resistance against spot blotch and CRR.

Currently, there is limited evidence that *Sb1* acts as an independent seedling resistance gene against *B. sorokiniana*. Reports of seedling-stage resistance associated with *Sb1*-linked markers are more plausibly explained by the presence of a broader resistance haplotype on chromosome 7DS, rather than by the direct action of *Lr34* itself. Markers such as *csLV34* are not functional markers of *Lr34* but tag a wider chromosomal region that may include linked minor-effect loci contributing to early disease responses.

In our study, resistance observed at the seedling stage was also consistently expressed at the adult plant stage, and statistical analysis confirmed a significant association between these phenotypes. Therefore, we interpret the seedling-stage responses as reflecting the contribution of the 7DS haplotype encompassing the *Sb1/Lr34* region rather than evidence that *Lr34* functions as a seedling resistance gene.

Previous studies have demonstrated that the spot blotch resistance locus *Sb1* is co-located with *Lr34* on chromosome 7DS and that this chromosomal region contributes to quantitative resistance to *B. sorokiniana* [48,72]. The resistance gene *Lr34* is classically characterized as an adult plant resistance (APR) gene and is therefore generally not expected to confer strong resistance at the seedling stage [49,71].

In the present study, although disease responses were initially recorded at the seedling stage, the same genotypes consistently expressed resistance at the adult plant stage, indicating continuity of resistance across developmental stages. These results suggest that the observed early-stage responses likely reflect the contribution of the broader 7DS resistance haplotype encompassing the *Sb1/Lr34* region, rather than classical seedling-specific resistance.

Our results indicate that *Sb1* and *Sb2* primarily contribute to resistance at the seedling stage, as evidenced by significantly lower infection scores in *Sb*-positive genotypes. The enhanced resistance observed in genotypes carrying both genes suggests an additive or partially synergistic genetic effect, which has also been reported in previous studies. However, the weaker expression of *Sb*-mediated resistance under field conditions highlights the stage-dependent nature of these genes and suggests that their effects are modulated by environmental factors and interactions with additional quantitative resistance loci.

Genotypes carrying both *Sb1* and *Sb2* showed the lowest seedling infection scores (≈0.8–1.0), suggesting an additive or synergistic effect, although the statistical advantage over single-gene carriers was not fully conclusive. Similar results have been reported in genetic studies showing that stacking multiple *Sb* loci can enhance resistance but often reaches a phenotypic plateau [44,46,48]. Notably, the strong effects of *Sb* genes at the seedling stage were less pronounced under field conditions, where several *Sb*-positive genotypes demonstrated only moderate adult plant resistance. Such discrepancies are well documented and may reflect environmental modulation of gene expression, interactions with additional QTL, or biological differences between foliar infections observed in seedling assays and root/crown infections occurring in the field [9,73,80].

Even so, several genotypes—particularly L-201m, #392/k-46619-Shenenskaya, #445/Chelyaba 80, #449/Orenburgskaya Yubileynaya, #456/k-38531-Albidum 43, and #459/k-43285-Saratovskaya 35—combined favorable *Sb* gene profiles with strong resistance at both seedling and adult stages. Their stable performance across years and under contrasting inoculation backgrounds makes them promising candidates for marker-assisted breeding programs, especially for wheat grown in continental climates like those of Kazakhstan.

While *Sb1* and *Sb2* play a key role in reducing early disease development, the observed field responses confirm that durable resistance to CRR requires the integration of *Sb* genes with additional quantitative loci. Therefore, *Sb*-linked markers represent an effective entry point for marker-assisted selection, but their use should be combined with multi-year field evaluations to achieve stable resistance.

Altogether, the phenotypic and molecular results demonstrate that CRR resistance is polygenic, partially stage-dependent, and strongly shaped by environmental variation. While *Sb1* and *Sb2* contribute significantly to seedling resistance, durable adult plant resistance likely depends on a broader set of quantitative loci whose combined effects must be considered during selection. Therefore, breeding strategies aimed at improving CRR resistance should integrate multi-year field evaluations, targeted use of *Sb*-linked markers, and pyramiding of quantitative resistance loci, which are increasingly emphasized in recent research [44,46,75,80]. Future work that incorporates genome-wide association studies, genomic prediction, and advanced root phenotyping technologies will help clarify the genetic architecture of CRR resistance and accelerate the development of wheat cultivars with durable and stable resistance.

## 5. Conclusions

This study demonstrated substantial genetic variation for resistance to common root rot in a diverse panel of wheat germplasm evaluated under natural, artificial, and fungicide-treated conditions. Leaf AUDPC proved to be a stable and informative indicator of quantitative resistance, whereas single-time severity scores varied strongly with annual environmental conditions. Molecular screening showed a high frequency of *Sb* genes, particularly *Sb2*, and confirmed their strong contribution to seedling resistance. Several genotypes combined favorable *Sb* gene profiles with consistent field resistance, identifying them as reliable donors for breeding.

The results highlight the polygenic nature of resistance and the need to integrate molecular markers with multi-year field testing to ensure accurate selection. The identified resistant genotypes and the generated dataset provide a solid basis for advancing breeding programs aimed at improving wheat adaptation and disease resilience in the dry regions of Kazakhstan.

## Figures and Tables

**Table 1:** Analysis of variance of the effects of genotype and year on field traits and genotype and replication on seedling resistance to *B. sorokiniana* in wheat.

Trait	Factor	SS	df	MS	F-Value	%SS	h^2^, %
Leaf AUDPC—field	Genotype	1530.56	155	98.745	18.42 ***	87.83	0.82
	Year	206.68	2	20.66	3.85 *	11.86	
	Residuals	5.359	310	53.598		0.30	
Field evaluation	Genotype	78,452.3	155	506.144	5.241 *	2.55	0.55
	Year	38,456.6	2	19,228.3	19.91 ***	96.9	
	Residuals	29,944.1	310	96.593		0.49	
*B. sorokiniana*—seedlings	Genotype	71.416	49	2.541	15.62 ***	31.56	0.63
	Replication	26.763	1	0.613	1.23	11.83	
	Residuals	125.10	49	0.254		55.29	

Notes: SS—sum of squares; df—degree of freedom; MS—mean squares; h^2^—broad-sense heritability index. *—significant difference at *p* < 0.05; ***—significant difference at *p* < 0.001.

**Table 2:** Genetic parameters in different resistance-related traits of wheat genotypes.

Trait	Min	Max	Mean	SE	Variance	SD	Median	Mode	CV
Leaf AUDPC 2023 (field)	10.5	693.0	218.17	14.326	34,018.62	178.93	185.75	21.0	82.01
Leaf AUDPC 2024 (field)	60.0	892.0	380.95	21.496	72,086.93	268.49	276.0	96.0	70.47
*B. sorokiniana* (seedlings)	0.8	4.6	2.20	0.166	1.44	1.19	1.7	1.6	54.52
Root severity (SCI—field)—tillering stage	0.0	19	6.21	0.521	44.46	6.51	5.0	0	104.80
Root severity (SCI—field)—milk stage	0.0	38	12.49	0.800	99.96	9.98	10.5	0	80.02
Root severity (SCI—field)—dough development stage	0.0	88	27.80	1.889	556.89	23.60	20.0	15.0	84.88

Notes: SD—standard deviation; SE—standard error; and CV—coefficient of variation.

**Table 3:** Pearson’s correlation matrix for disease severity scores, AUDPC values, and seedling resistance to *B. sorokiniana.*

Variables, Disease Severity	Tillering Stage, %	Milk Stage, %	Dough Development Stage, %	Leaf AUDPC 2023 (Field)	Leaf AUDPC 2024 (Field)	*B. sorokiniana* (Seedlings)
**T** **illering stage, %**	1	0.762	0.737	0.634	0.511	−0.306
**Milk stage, %**	0.762	1	0.840	0.666	0.560	−0.403
**Dough development stage, %**	0.737	0.840	1	0.625	0.561	−0.372
**Leaf AUDPC 2023 (Field)**	0.634	0.666	0.625	1	0.948	−0.247
**Leaf AUDPC 2024 (Field)**	0.511	0.560	0.561	0.948	1	−0.184
***B. sorokiniana* (seedlings)**	−0.306	−0.403	−0.372	−0.247	−0.184	1

## Data Availability

The data presented in this study are available on request from the corresponding author. The data is publicly available.

## Supplementary Materials

The following supporting information can be downloaded at: https://www.mdpi.com/article/10.3390/biology15030244/s1. Table S1: Disease severity for *B. sorokiniana* in the Aktobe region and sources of *Sb* genes based on linked markers in the wheat genotypes; Table S2: Molecular markers used to identify *Sb* genes; Table S3: Distribution of wheat accessions by seedling resistance levels to *B. sorokiniana*; Table S4: Distribution of wheat genotypes by level of resistance to common root rot based on AUDPC values; Figure S1: Distribution of accessions by resistance levels *B. sorokiniana*; Figure S2: Impact of *Sb* genes *(Sb1*, *Sb2*, *Sb1* + *Sb2)* on wheat seedling resistance to *B. sorokiniana*; Figure S3: Descriptive statistics for the evaluated population.
