# Supplementary material for: Molecular Marker-Based Identification of Resistance to Bipolaris sorokiniana in Kazakh and Global Wheat Germplasm
Source: Biology (Basel). 2026 Jan 28;15(3):244. doi: 10.3390/biology15030244 (PMC12897019; doi:10.3390/biology15030244)
Supplement: Supplementary file 1 [file biology-15-00244-s001.zip › Supplementary Table S4.pdf]

Supplementary Table S4. Distribution of wheat genotypes by level of resistance to common root rot based on AUDPC values

Background, year	Wheat entries with a high level of disease resistance	Wheat entries with a moderate level of disease resistance	Wheat entries with a susceptible level of disease resistance
Natural, 2023	9 samples: #363/k-43130 (52); #377/k-44889-Yogui (63); #576/k-41350-Krasnokutka (70); #354/k-41295-Bankuti Garnet (78); #577/k-51744-Melanopus 2824 (80,5); #445/Chelyaba 80 (84); L-201m (98); #322/k-30949 (98); Kargala 9 (98)	35 samples: #392/k-46619-Shenenskaya (101,5); #459/k-43285-Saratovskaya 35 (101,5); #544/Gordeya (105); #660/Line-2285-d.3 (108,5); #324/k-31833 (112); #418/k-64467-Baganskaya 93 (112); #449/Orenburgskaya Yubileynaya (115,5); #464/k-54045-Tselinnaya 21 (115,5); #308/k-12927-delfi (119); #378/k-45151-Oktavia (122,5); #366/k-43878 (126); #306/k-17172-Salamush (129,5); #456/k-38531-Albidum 43 (129,5); #439/Silantiy (133); #507/k-64721-Gord. 1732 (185,5); #553/Gordeiforme-00-171-4 (185,5); #643/Gordeiforme-2441 (186); #450/Silach (189); #581/Prinadur (189); #531/Gordeiforme-910 (192,5); #644/Gordeiforme-2246 (196); #352/k-40599-Saratovskaya 29 (206,5); #523/Kostanayskaya 15 (206,5); #347/k-38532-Albidum 24 (210); #655/G-13-62-2 (210); #517/Seymour 16 (210); #303/k-12589 (220,5); #555/Elizavetinskaya (220,5); #407/k-52321-WW16628 (224); #316/k-25761 (227,5); #362/к-43109-Licofen (227,5); #524/Gordeiforme 1790 (227,5); #318/k-28130-Smena (231); #508/k-64723-Leucurum 1751 (238); #317/k-28117-Blancar (248,5)	6 samples: #573/k-64967-Orenburgskaya 21 (301); #506/k-64718-Gord. 1739 (308); L-248/258 (312); #567/Tselinogradskaya 75 (325); #527/Bezenchukskay 139 (332); #353/k-41218-Saratovskaya 28 (368)
Natural, 2024	8 samples: #363/k-43130 (158); #377/k-44889-Yogui (178); #576/k-41350-Krasnokutka (180); #577/k-51744-Melanopus 2824 (186); #445/Chelyaba 80 (192); #449/Orenburgskaya Yubileynaya (196); #392/k-46619-Shenenskaya (198); #459/k-43285-Saratovskaya 35 (198)	35 samples: L-201m (204); #322/k-30949 (204); #354/k-41295-Bankuti Garnet (210); #660/Line-2285-d.3 (210); #544/Gordeya (215); #308/k-12927-delfi (216); #324/k-31833 (216); #418/k-64467-Baganskaya 93 (216); #378/k-45151-Oktavia (222); #464/k-54045-Tselinnaya 21 (222); #366/k-43878 (228); #306/k-17172-Salamush (234); #456/k-38531-Albidum 43 (234); #439/Silantiy (240); Kargala 9 (244); #507/k-64721-Gord. 1732 (270); #450/Silach (276); #531/Gordeiforme-910 (276); #553/Gordeiforme-00-171-4 (276); #352/k-40599-Saratovskaya 29 (288); #508/k-64723-Leucurum 1751 (288); #303/k-12589 (294); #317/k-28117-Blancar (294); #347/k-38532-Albidum 24 (294); #517/Seymour 16 (294); #581/Prinadur (294); #655/G-13-62-2 (294); #362/к-43109-Licofen (300); #316/k-25761 (306); #318/k-28130-Smena (306); #407/k-52321-WW16628 (306); #555/Elizavetinskaya (338); #643/Gordeiforme-2441 (345); #644/Gordeiforme-2246 (347); #524/Gordeiforme 1790 (348)	7 samples: #523/Kostanayskaya 15 (388); #573/k-64967-Orenburgskaya 21 (448); #506/k-64718-Gord. 1739 (458); #527/Bezenchukskay 139 (458); #353/k-41218-Saratovskaya 28 (494); L-248/258 (500); #567/Tselinogradskaya 75 (500)

Fungicidal, 2023	23 samples: #322/k-30949 (10,5); #377/k-44889-Yogui (10,5); #378/k-45151-Oktavia (10,5); #418/k-64467-Baganskaya 93 (10,5); L-201m (14); #306/k-17172-Salamush (17,5); #324/k-31833 (17,5); #366/k-43878 (17,5); #459/k-43285-Saratovskaya 35 (17,5); #354/k-41295-Bankuti Garnet (21); #392/k-46619-Shenenskaya (21); #445/Chelyaba 80 (21); #449/Orenburgskaya Yubileynaya (21); #464/k-54045-Tselinnaya 21 (21); Kargala 9 (21); #576/k-41350-Krasnokutka (21); #577/k-51744-Melanopus 2824 (21); #660/Line-2285-d.3 (21); #363/k-43130 (24,5); #439/Silantiy (24,5); #456/k-38531-Albidum 43 (24,5); #308/k-12927-delfi (28); #544/Gordeya (28)	24 samples: #555/Elizavetinskaya (52,5); #553/Gordeiforme-00-171-4 (66,5); #303/k-12589 (73,5); #523/Kostanayskaya 15 (73,5); #567/Tselinogradskaya 75 (73,5); #507/k-64721-Gord. 1732 (77); #524/Gordeiforme 1790 (77); #573/k-64967-Orenburgskaya 21 (77); #581/Prinadur (77); #643/Gordeiforme-2441 (77); #644/Gordeiforme-2246 (77); #316/k-25761 (80,5); #317/k-28117-Blancar (80,5); #318/k-28130-Smena (80,5); #353/k-41218-Saratovskaya 28 (80,5); #362/к-43109-Licofen (80,5); #407/k-52321-WW16628 (80,5); #517/Seymour 16 (80,5); #347/k-38532-Albidum 24 (87,5); #450/Silach (87,5); #655/G-13-62-2 (87,5); #527/Bezenchukskay 139 (91); #352/k-40599-Saratovskaya 29 (94,5); #531/Gordeiforme-910 (98)	3 samples: #508/k-64723-Leucurum 1751 (112); L-248/258 (122,5); #506/k-64718-Gord. 1739 (150,5)
Fungicidal, 2024	18 samples: #322/k-30949 (78); #377/k-44889-Yogui (78); #378/k-45151-Oktavia (78); #418/k-64467-Baganskaya 93 (78); L-201m (84); #306/k-17172-Salamush (90); #324/k-31833 (90); #366/k-43878 (90); #354/k-41295-Bankuti Garnet (96); #392/k-46619-Shenenskaya (96); #445/Chelyaba 80 (96); #449/Orenburgskaya Yubileynaya (96); #459/k-43285-Saratovskaya 35 (96); #464/k-54045-Tselinnaya 21 (96); Kargala 9 (96); #576/k-41350-Krasnokutka (96); #577/k-51744-Melanopus 2824 (96); #660/Line-2285-d.3 (96)	30 samples: #363/k-43130 (102); #439/Silantiy (102); #456/k-38531-Albidum 43 (102); #308/k-12927-delfi (108); #544/Gordeya (108); #655/G-13-62-2 (150); #450/Silach (156); #553/Gordeiforme-00-171-4 (156); #573/k-64967-Orenburgskaya 21 (156); #581/Prinadur (156); #643/Gordeiforme-2441 (156); #555/Elizavetinskaya (162); #318/k-28130-Smena (162); #524/Gordeiforme 1790 (162); #316/k-25761 (168); #317/k-28117-Blancar (168); #407/k-52321-WW16628 (168); #506/k-64718-Gord. 1739 (168); #507/k-64721-Gord. 1732 (168); #523/Kostanayskaya 15 (168); #527/Bezenchukskay 139 (168); #567/Tselinogradskaya 75 (168); #347/k-38532-Albidum 24 (174); #352/k-40599-Saratovskaya 29 (174#644/Gordeiforme-2246 (174); #517/Seymour 16 (180); #531/Gordeiforme-910 (180); #303/k-12589 (186); #353/k-41218-Saratovskaya 28 (186); #508/k-64723-Leucurum 1751 (186); L-248/258 (192)	2 samples:): #362/к-43109-Licofen (174); L-248/258 (192)
Infectious, 2023	5 samples: #366/k-43878 (213,5); #378/k-45151-Oktavia (224); L-201m (252); #306/k-17172-Salamush (254); #418/k-64467-Baganskaya 93 (255,5)	23 samples: #324/k-31833 (305); #449/Orenburgskaya Yubileynaya (307); #322/k-30949 (308); #439/Silantiy (308); #464/k-54045-Tselinnaya 21 (308); #445/Chelyaba 80 (312); Kargala 9 (312); #354/k-41295-Bankuti Garnet (318); #377/k-44889-Yogui (318); #577/k-51744-Melanopus 2824 (318); #544/Gordeya (324); #456/k-38531-Albidum 43 (325); #308/k-	22 samples: #655/G-13-62-2 (504); #644/Gordeiforme-2246 (507,5); #353/k-41218-Saratovskaya 28 (511); #352/k-40599-Saratovskaya 29 (514,5); #523/Kostanayskaya 15 (518);

		12927-delfi (328); #555/Elizavetinskaya (333); #459/k-43285-Saratovskaya 35 (343); #576/k-41350-Krasnokutka (343); #363/k-43130 (345); #392/k-46619-Shenenskaya (345); #660/Line-2285-d.3 (365); #567/Tselinogradskaya 75 (427); #517/Seymour 16 (430,5); #531/Gordeiforme-910 (458,5); #573/k-64967-Orenburgskaya 21 (458,5)	#581/Prinadur (528,5); #318/k-28130-Smena (528,5); #407/k-52321-WW16628 (532); #507/k-64721-Gord. 1732 (532); #524/Gordeiforme 1790 (542,5); #303/k-12589 (546); #317/k-28117-Blancar (546); #553/Gordeiforme-00-171-4 (560); #316/k-25761 (570,5); #362/к-43109-Licofen (570,5); #508/k-64723-Leucurum 1751 (581); #450/Silach (584); L-248/258 (591,5); #527/Bezenchukskay 139 (595); #506/k-64718-Gord. 1739 (598); #643/Gordeiforme-2441 (612); #347/k-38532-Albidum 24 (663)
Infectious, 2024	1 sample: #363/k-43130 (492)	23 samples: #366/k-43878 (544); #377/k-44889-Yogui (565); #445/Chelyaba 80 (568); #354/k-41295-Bankuti Garnet (582); #418/k-64467-Baganskaya 93 (582); #306/k-17172-Salamush (584); L-201m (585); #544/Gordeya (586); #324/k-31833 (589); #459/k-43285-Saratovskaya 35 (604); #392/k-46619-Shenenskaya (605); #439/Silantiy (614); #449/Orenburgskaya Yubileynaya (614); #308/k-12927-delfi (618); #456/k-38531-Albidum 43 (618); #577/k-51744-Melanopus 2824 (618); Kargala 9 (620); #322/k-30949 (624); #660/Line-2285-d.3 (644); #378/k-45151-Oktavia (645); #464/k-54045-Tselinnaya 21 (645); #576/k-41350-Krasnokutka (666); #555/Elizavetinskaya (674)	26 samples: #352/k-40599-Saratovskaya 29 (784); #507/k-64721-Gord. 1732 (786); #531/Gordeiforme-910 (787); #517/Seymour 16 (788); #318/k-28130-Smena (796); #644/Gordeiforme-2246 (799); #573/k-64967-Orenburgskaya 21 (802); #353/k-41218-Saratovskaya 28 (812); #362/к-43109-Licofen (814); #523/Kostanayskaya 15 (815); #316/k-25761 (832); #508/k-64723-Leucurum 1751 (845); #553/Gordeiforme-00-171-4 (845); #317/k-28117-Blancar (847); #581/Prinadur (848); #655/G-13-62-2 (858); #506/k-64718-Gord. 1739 (862); L-248/258 (868); #643/Gordeiforme-2441 (868); #407/k-52321-WW16628 (884); #567/Tselinogradskaya 75 (884); #303/k-12589 (885); #524/Gordeiforme 1790 (885); #450/Silach (886); #527/Bezenchukskay 139 (892); #347/k-38532-Albidum 24 (892)
