# Supplementary material for: Molecular Marker-Based Identification of Resistance to Bipolaris sorokiniana in Kazakh and Global Wheat Germplasm
Source: Biology (Basel). 2026 Jan 28;15(3):244. doi: 10.3390/biology15030244 (PMC12897019; doi:10.3390/biology15030244)

Supplementary Figure S1. Distribution of accessions by resistance levels *Bipolaris sorokiniana*

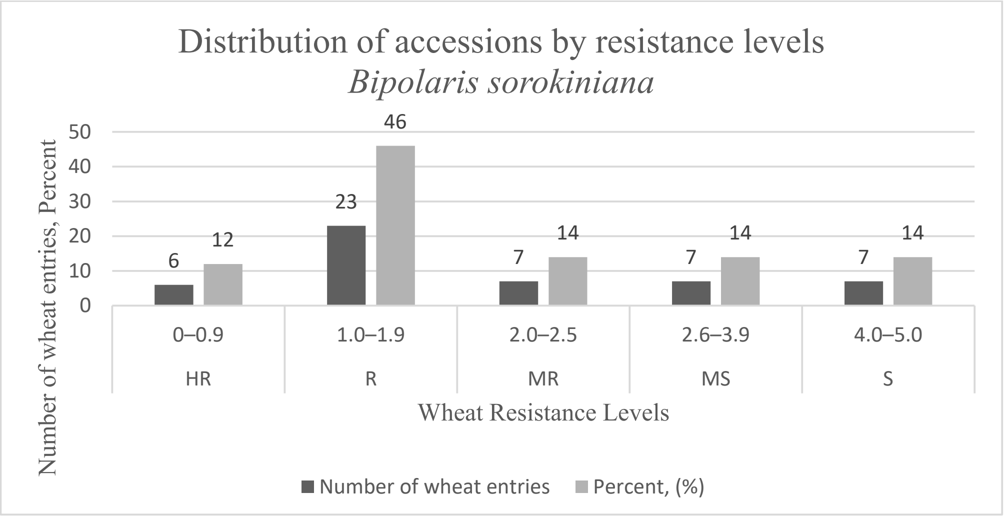

Supplement: Supplementary file 1 [file biology-15-00244-s001.zip › Supplementary Figure S1.pdf]
