# Supplementary material for: Molecular Marker-Based Identification of Resistance to Bipolaris sorokiniana in Kazakh and Global Wheat Germplasm
Source: Biology (Basel). 2026 Jan 28;15(3):244. doi: 10.3390/biology15030244 (PMC12897019; doi:10.3390/biology15030244)
Supplement: Supplementary file 1 [file biology-15-00244-s001.zip › Supplementary Figure S2.pdf]

Supplementary Figure S2. Impact of *Sb* genes *(Sb1, Sb2, Sb1+Sb2)* on wheat seedling resistance to *B. sorokiniana*

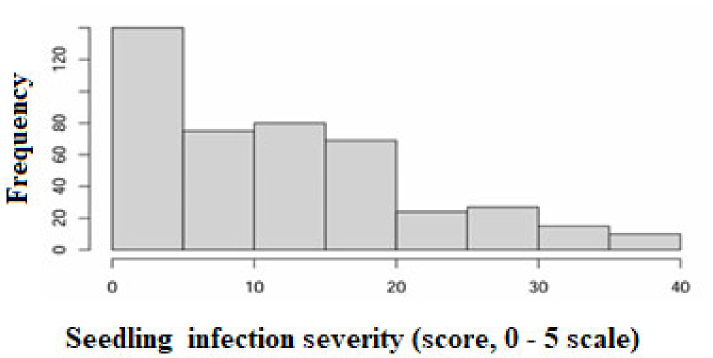

Genes	*B.sorokiniana* (seedlings)
none	3,95
Sb1	1,40
Sb1, Sb2	0,83
Sb2	1,61
p-value	<0.01
