# Supplementary material for: Molecular Marker-Based Identification of Resistance to Bipolaris sorokiniana in Kazakh and Global Wheat Germplasm
Source: Biology (Basel). 2026 Jan 28;15(3):244. doi: 10.3390/biology15030244 (PMC12897019; doi:10.3390/biology15030244)
Supplement: Supplementary file 1 [file biology-15-00244-s001.zip › Supplementary Figure S3.pdf]

Supplementary Figure S3. Descriptive statistics for the evaluated population

Min.	1st Qu.	Median	Mean	3rd Qu.	Max.
0.00	3.00	11.00	11.79	17.25	38.00
