# Supplementary material for: Molecular Marker-Based Identification of Resistance to Bipolaris sorokiniana in Kazakh and Global Wheat Germplasm
Source: Biology (Basel). 2026 Jan 28;15(3):244. doi: 10.3390/biology15030244 (PMC12897019; doi:10.3390/biology15030244)
Supplement: Supplementary file 1 [file biology-15-00244-s001.zip › Supplementary Table S1.pdf]

Supplementary Table S1. Disease severity for *B. sorokiniana* in the Aktobe region and sources of *Sb* genes based on linked markers in the wheat genotypes.

Name of wheat	Origin	Background	Field score across developmental stages, %				Leaf AUDPC 2023 (Field)	Leaf AUDPC 2024 (Field)	*B.sorokiniana* (seedlings)	Presence *Sb* genes		
			tillering	milk	dough development	Root Disease Severity (DS), %				*Sb1*	*Sb2*	Total *Sb* genes
**Common wheat (*Triticum aestivum*)**												
L-201m	RK, Aktobe Agricultural Experimental Station	Natural infection	1	7	13	21,25	98	204	0,9	+	+	*Sb1, Sb2*
		Fungicide-treated	0	0	4	8,75	14	84				
		Artificial inoculation	12	18	24	34,17	252	585				
#303/k-12589	Afghanistan	Natural infection	7	16	24	31,25	220,5	294	1,8	-	+	*Sb2*
		Fungicide-treated	0	3	15	18,33	73,5	186				
		Artificial inoculation	18	28	82	45,42	546	885				
#306/k-17172-Salamush	Syria	Natural infection	3	9	16	24,17	129,5	234	4,3	-	-	*none*
		Fungicide-treated	0	0	5	9,17	17,5	90				
		Artificial inoculation	14	20	32	37,50	254	584				
#308/k-12927-delfi	Türkiye	Natural infection	4	9	12	22,92	119	216	3,8	-	-	*none*
		Fungicide-treated	0	0	8	11,25	28	108				
		Artificial inoculation	16	20	28	37,50	328	618				

#316/k-25761	Kyrgyzstan	Natural infection	9	16	24	32,08	227,5	306	1,6	-	+	*Sb2*
		Fungicide-treated	0	4	15	17,50	80,5	168				
		Artificial inoculation	17	32	82	45,83	570,5	832				
#317/k-28117-Blancar	Russia, Saratov region	Natural infection	17	15	24	30,83	248,5	294	1,4	-	+	*Sb2*
		Fungicide-treated	0	4	15	17,50	80,5	168				
		Artificial inoculation	18	28	82	45,42	546	847				
#318/k-28130-Smena	Russia, Omsk region	Natural infection	8	16	26	32,08	231	306	1,9	-	+	*Sb2*
		Fungicide-treated	0	5	13	17,50	80,5	162				
		Artificial inoculation	17	26	82	44,58	528,5	796				
#322/k-30949	Russia, Tula region	Natural infection	4	7	10	21,25	98	204	4,2	-	-	*none*
		Fungicide-treated	0	0	3	7,92	10,5	78				
		Artificial inoculation	11	18	34	35,83	308	624				
#324/k-31833	Tajikistan	Natural infection	1	8	15	22,50	112	216	3,9	-	-	*none*
		Fungicide-treated	0	0	5	9,58	17,5	90				
		Artificial inoculation	9	15	24	31,67	305	589				
#347/k-38532-Albidum 24	Russia, Saratov region	Natural infection	6	14	26	30,42	210	294	1,9	-	+	*Sb2*
		Fungicide-treated	0	4	17	17,92	87,5	174				
		Artificial inoculation	17	32	58	44,17	663	892				
#352/k-40599-Saratovskaya 29	Russia, Saratov region	Natural infection	5	14	26	30,00	206,5	288	1,6	-	+	*Sb2*
		Fungicide-treated	0	6	15	18,75	94,5	174				
		Artificial inoculation	17	30	70	44,58	514,5	784				
#353/k-41218-Saratovskaya 28	Russia, Saratov region	Natural infection	7	16	24	31,25	368	494	1,8	-	+	*Sb2*
		Fungicide-treated	0	4	15	18,75	80,5	186				
		Artificial inoculation	16	24	82	43,75	511	812				
	Hungary	Natural infection	1	8	14	22,08	78	210	3,8	-	-	*none*

#354/k-41295-Bankuti Garnet		Fungicide-treated	0	0	6	10,00	21	96				
		Artificial inoculation	11	19	34	36,25	318	582				
#362/к-43109-Licofen	Chile	Natural infection	7	16	26	31,67	227,5	300	1,5	-	+	*Sb2*
		Fungicide-treated	0	4	15	17,92	80,5	222				
		Artificial inoculation	17	38	70	45,83	570,5	814				
#363/k-43130	Peru	Natural infection	1	8	13	21,67	52	158	1,4	+	-	*Sb1*
		Fungicide-treated	0	0	7	10,42	24,5	102				
		Artificial inoculation	12	19	36	37,08	345	492				
#366/k-43878	India	Natural infection	2	9	16	23,75	126	228	2,8	-	-	*none*
		Fungicide-treated	0	0	5	9,17	17,5	90				
		Artificial inoculation	11	15	20	32,08	213,5	544				
#377/k-44889-Yogui	USA	Natural infection	3	0	15	18,33	63	178	3,7	-	-	*none*
		Fungicide-treated	0	0	3	8,33	10,5	78				
		Artificial inoculation	11	18	28	34,58	318	565				
#378/k-45151-Oktavia	Czechoslovakia	Natural infection	1	9	16	23,33	122,5	222	3,4	-	-	*none*
		Fungicide-treated	0	0	3	8,33	10,5	78				
		Artificial inoculation	10	15	24	32,08	224	645				
#392/k-46619-Shenenskaya	Russia, Smolensk region	Natural infection	1	8	12	21,25	101,5	198	0,8	+	+	*Sb1, Sb2*
		Fungicide-treated	0	0	6	10,00	21	96				
		Artificial inoculation	1,2	18	32	35,83	345	605				
#407/k-52321-WW16628	Sweden	Natural infection	6	14	30	31,25	224	306	1,6	-	+	*Sb2*
		Fungicide-treated	0	4	15	17,50	80,5	168				
		Artificial inoculation	16	24	88	44,17	532	884				
#418/k-64467-Baganskaya 93	Russia, Novosibirsk region	Natural infection	3	8	13	22,50	112	216	3,8	-	-	*none*
		Fungicide-treated	0	0	3	8,33	10,5	78				

		Artificial inoculation	11	18	26	34,17	255,5	582				
#439/Silantiy	Russia, Omsk State Agrarian University	Natural infection	4	9	16	24,58	133	240	4,2	-	-	*none*
		Fungicide-treated	0	0	7	10,83	24,5	102				
		Artificial inoculation	11	19	36	36,67	308	614				
#445/Chelyaba 80	Russia, Chelyabinsk. Research Institute of Agriculture	Natural infection	2	6	10	20,00	84	192	0,8	+	+	*Sb1, Sb2*
		Fungicide-treated	0	0	6	10,00	21	96				
		Artificial inoculation	11	18	34	35,83	312	568				
#449/Orenburgskaya Yubileynaya	Russia, Orenburg Research Institute of Agriculture	Natural infection	3	9	12	22,50	115,5	196	0,8	+	+	*Sb1, Sb2*
		Fungicide-treated	0	0	6	10,00	21	96				
		Artificial inoculation	12	19	34	36,67	307	614				
#450/Silach	Russia, Chelyabinsk. Research Institute of Agriculture	Natural infection	6	14	20	29,17	189	276	1,3	-	+	*Sb2*
		Fungicide-treated	0	6	13	17,50	87,5	156				
		Artificial inoculation	17	24	76	43,75	584	886				
#456/k-38531-Albidum 43	Russia, Saratov region	Natural infection	3	9	16	24,17	129,5	234	0,9	+	+	*Sb1, Sb2*
		Fungicide-treated	0	0	7	10,42	24,5	102				
		Artificial inoculation	12	18	32	35,83	325	618				
#459/k-43285-Saratovskaya 35	Russia, Saratov region	Natural infection	1	8	12	21,25	101,5	198	0,8	+	+	*Sb1, Sb2*
		Fungicide-treated	0	0	5	10,00	17,5	96				
		Artificial inoculation	12	22	34	37,50	343	604				
#464/k-54045-Tselinnaya 21	Kazakhstan, Barayev Scientific Production Center for Grain Farming	Natural infection	2	8	15	22,92	115,5	222	1,6	-	+	*Sb2*
		Fungicide-treated	0	0	6	10,42	21	96				
		Artificial inoculation	14	20	34	37,92	308	645				
**Durum wheat ((*Triticum durum*)**												
Kargala 9	RK, Aktobe Agricultural Experimental Station	Natural infection	0	8	12	20,83	98	244	4,2	-	-	*none*
		Fungicide-treated	0	0	6	10,00	21	96				

		Artificial inoculation	11	20	36	37,08	312	620				
L-248/258	RK, Aktobe Agricultural Experimental Station	Natural infection	6	14	26	30,42	312	500	1,5	-	+	*Sb2*
		Fungicide-treated	0	9	17	21,25	122,5	236				
		Artificial inoculation	17	38	76	46,25	591,5	868				
#506/k-64718-Gord. 1739	Russia	Natural infection	9	18	22	32,50	308	458	1,9	-	+	*Sb2*
		Fungicide-treated	0	14	15	17,50	150,5	168				
		Artificial inoculation	16	32	58	43,75	598	862				
#507/k-64721-Gord. 1732	Russia	Natural infection	5	14	20	28,75	185,5	270	1,3	-	+	*Sb2*
		Fungicide-treated	0	4	14	17,50	77	168				
		Artificial inoculation	18	32	70	45,42	532	786				
#508/k-64723-Leucurum 1751	Russia	Natural infection	6	19	24	32,08	238	288	1,8	-	+	*Sb2*
		Fungicide-treated	0	7	18	20,00	112	186				
		Artificial inoculation	18	36	76	46,25	581	845				
#517/Seymour 16	Kazakhstan. KazNIIZIR	Natural infection	8	15	22	30,83	210	294	1,6	-	+	*Sb2*
		Fungicide-treated	0	4	15	18,33	80,5	180				
		Artificial inoculation	17	24	58	42,50	430,5	788				
#523/Kostanayskaya 15	Kazakhstan, Karabalyk Agricultural Experimental Station	Natural infection	5	14	26	30,00	206,5	388	1,5	-	+	*Sb2*
		Fungicide-treated	0	3	15	17,08	73,5	168				
		Artificial inoculation	16	28	76	44,17	518	815				
#524/Gordeiforme 1790	Kazakhstan, Karabalyk Agricultural Experimental Station	Natural infection	7	15	28	31,67	227,5	348	1,0	-	+	*Sb2*
		Fungicide-treated	0	4	14	17,08	77	162				
		Artificial inoculation	15	38	64	44,58	542,5	885				
#527/Bezenchukskay 139	Russia, Samara Research Institute of Agriculture	Natural infection	7	18	30	33,33	332	458	1,8	-	+	*Sb2*
		Fungicide-treated	0	6	14	18,33	91	168				

		Artificial inoculation	18	32	88	46,67	595	892					
#531/Gordeiforme-910	Russia, Altai Research Institute of Agriculture	Natural infection	5	14	22	29,17	192,5	276	1,6	-	+	*Sb2*	
		Fungicide-treated	0	6	16	19,17	98	180					
		Artificial inoculation	15	26	64	42,50	458,5	787					
#544/Gordeya	Russia, Orenburg Research Institute of Agriculture	Natural infection	0	8	14	21,67	105	215	4,6	-	-	*none*	
		Fungicide-treated	0	0	8	10,83	28	108					
		Artificial inoculation	9	14	24	31,25	324	586					
#553/Gordeiforme-00-171-4	Russia, Omsk Research Institute of Agriculture	Natural infection	5	13	22	28,75	185,5	276	1,3	-	+	*Sb2*	
		Fungicide-treated	0	2	15	15,83	66,5	156					
		Artificial inoculation	16	28	88	45,00	560	845					
#555/Elizavetinskaya	Russia, Saratov Research Institute of Agriculture	Natural infection	9	14	26	31,67	220,5	338	4,3	-	-	*none*	
		Fungicide-treated	0	0	15	15,42	52,5	162					
		Artificial inoculation	18	32	76	45,83	333	674					
#567/Tselinogradskaya 75	Kazakhstan, Barayev Scientific Production Center for Grain Farming	Natural infection	5	100	28	33,75	325	500	1,4	-	+	*Sb2*	
		Fungicide-treated	0	3	15	17,08	73,5	168					
		Artificial inoculation	16	24	58	42,08	427	884					
#573/k-64967-Orenburgskaya 21	Russia, Orenburg Research Institute of Agriculture	Natural infection	7	18	24	32,08	301	448	1,1	-	+	*Sb2*	
		Fungicide-treated	0	4	14	16,67	77	156					
		Artificial inoculation	19	24	64	43,75	458,5	802					
#576/k-41350-Krasnokutka	Russia, Saratov region	Natural infection	0	5	10	18,75	70	180	4,5	-	-	*none*	
		Fungicide-treated	0	0	6	10,00	21	96					
		Artificial inoculation	11	19	36	36,67	343	666					
#577/k-51744-Melanopus 2824	Russia, Saratov region	Natural infection	1	6	10	19,58	80,5	186	1,2	-	+	*Sb2*	
		Fungicide-treated	0	0	6	10,00	21	96					

		Artificial inoculation	12	22	36	37,92	318	618				
#581/Prinadur	France	Natural infection	6	11	26	29,17	189	294	1,6	-	+	*Sb2*
		Fungicide-treated	0	4	14	16,67	77	156				
		Artificial inoculation	17	26	82	44,58	528,5	848				
#643/Gordeiforme-2441	Kazakhstan, Karabalyk Agricultural Experimental Station	Natural infection	7	16	26	31,67	186	345	1,8	-	+	*Sb2*
		Fungicide-treated	0	4	14	16,67	77	156				
		Artificial inoculation	16	24	64	42,50	612	868				
#644/Gordeiforme-2246	Kazakhstan, Karabalyk Agricultural Experimental Station	Natural infection	8	14	20	30,00	196	347	1,5	-	+	*Sb2*
		Fungicide-treated	0	3	16	17,50	77	174				
		Artificial inoculation	17	26	76	44,17	507,5	799				
#655/G-13-62-2	Russia, Omsk Agricultural Research Center	Natural infection	6	14	26	30,42	210	294	1,8	-	+	*Sb2*
		Fungicide-treated	0	6	13	17,08	87,5	150				
		Artificial inoculation	16	26	76	43,75	504	858				
#660/Line-2285-d.3	Russia, Samara Research Institute of Agriculture	Natural infection	0	8	15	22,08	108,5	210	1,9	-	+	*Sb2*
		Fungicide-treated	0	0	6	10,00	21	96				
		Artificial inoculation	10	19	36	36,25	365	644				
Salaumoni	Lebanon	Natural infection	0	0	0	5,00	324	450	3,8	-	-	*none*
		Fungicide-treated	0	1	1	4,17	17	60				
		Artificial inoculation	0	6	12	13,33	514	866				
Gleanlea	Canada	Natural infection	6	10	24	8,33	82	196	1,8	-	+	*Sb2*
		Fungicide-treated	0	6	12	6,67	36	152				
		Artificial inoculation	14	18	84	15,83	218	468				

Notes: According to field evaluations, disease severity at different developmental stages was categorized as follows: 0 – 10% - highly resistant (HR), 11 – 20% - resistant (R), 21 – 40% - moderately susceptible (MS), and 41 – 100% - susceptible (S) [58]. Overall field resistance was assessed based on the area under the disease progress curve (AUDPC) calculated for the 2023 and 2024 growing seasons. Seedling responses were rated on a 0 – 5 scale [59], where 0 – 0,9 corresponds to highly resistant (HR); 1,0 – 1,9 to resistant (R); 2,0

– 2,5 to moderately resistant (MR); 2,6 – 3,9 to moderately susceptible (MS); 4,0 – 5,0 to susceptible (S). "B. sorokiniana (seedling)" refers to the response of seedlings to inoculation with a mixture of *B. sorokiniana* pathotypes. The presence of Sb genes conferring resistance to common root rot was determined in the wheat genotype collection using linked molecular markers: *Sb1*(STS marker *csLV34*) and *Sb2* (SSR marker *Xfcp623*).
