# Supplementary material for: Molecular Marker-Based Identification of Resistance to Bipolaris sorokiniana in Kazakh and Global Wheat Germplasm
Source: Biology (Basel). 2026 Jan 28;15(3):244. doi: 10.3390/biology15030244 (PMC12897019; doi:10.3390/biology15030244)
Supplement: Supplementary file 1 [file biology-15-00244-s001.zip › Supplementary Table S2.pdf]

Supplementary Table S2. Molecular markers used to identify *Sb* genes

№	**Gen**	**Chr**	**Type of Marker**	**Primer name**	**Sequence of Primers 5'-3'**	**Anneling Temperature, °C**	**Fragment Size, bp**	**Reference**
1	*Sb1*	7DS	STS	*csLV34*	5'- GTT GGT TAA GAC TGG TGA TGG-3'<br>5'-TGC TTG CTA TTG CTG AAT AGT-3'	55°C	+150bp.	Lagudah et al., 2009 [71]
2	*Sb2*	5BL	SSR	*Xfcp623*	5'- CTA TTC GTA ATC GTG CCT TCC G -3'<br>5'- CCT TCT CTC TCA CCG CTA TCT CAT C -3'	60°C	+380bp.	Lillemo et al, 2013 [48]<br>Zhang et al, 2009 [51]
