# Supplementary material for: Molecular Marker-Based Identification of Resistance to Bipolaris sorokiniana in Kazakh and Global Wheat Germplasm
Source: Biology (Basel). 2026 Jan 28;15(3):244. doi: 10.3390/biology15030244 (PMC12897019; doi:10.3390/biology15030244)
Supplement: Supplementary file 1 [file biology-15-00244-s001.zip › Supplementary Table S3.pdf]

Supplementary Table S3. Distribution of wheat accessions by seedling resistance levels to *B. sorokiniana*

Sustainability category	Disease severity	Number of wheat entries	%
Highly resistant (HR)	0 – 0.9	6	12,00
Resistance (R)	1.0 – 1.9	23	46,00
Moderately Resistant (MR)	2.0 – 2.5	7	14,00
Moderately Susceptible (MS)	2.6 – 3.9	7	14,00
Susceptible (S)	4.0 – 5.0	7	14,00
